# DNA backbone interactions impact the sequence specificity of DNA sulfur-binding domains: revelations from structural analyses

**DOI:** 10.1093/nar/gkaa574

**Published:** 2020-07-04

**Authors:** Hao Yu, Jiayi Li, Guang Liu, Gong Zhao, Yuli Wang, Wenyue Hu, Zixin Deng, Geng Wu, Jianhua Gan, Yi-Lei Zhao, Xinyi He

**Affiliations:** State Key Laboratory of Microbial Metabolism, Joint International Research Laboratory of Metabolic & Developmental Sciences, School of Life Sciences & Biotechnology, Shanghai Jiao Tong University, 800 Dongchuan Road, Shanghai 200240, People's Republic of China; State Key Laboratory of Microbial Metabolism, Joint International Research Laboratory of Metabolic & Developmental Sciences, School of Life Sciences & Biotechnology, Shanghai Jiao Tong University, 800 Dongchuan Road, Shanghai 200240, People's Republic of China; State Key Laboratory of Bioreactor Engineering, East China University of Science and Technology, Shanghai 200237, People's Republic of China; State Key Laboratory of Microbial Metabolism, Joint International Research Laboratory of Metabolic & Developmental Sciences, School of Life Sciences & Biotechnology, Shanghai Jiao Tong University, 800 Dongchuan Road, Shanghai 200240, People's Republic of China; State Key Laboratory of Microbial Metabolism, Joint International Research Laboratory of Metabolic & Developmental Sciences, School of Life Sciences & Biotechnology, Shanghai Jiao Tong University, 800 Dongchuan Road, Shanghai 200240, People's Republic of China; State Key Laboratory of Microbial Metabolism, Joint International Research Laboratory of Metabolic & Developmental Sciences, School of Life Sciences & Biotechnology, Shanghai Jiao Tong University, 800 Dongchuan Road, Shanghai 200240, People's Republic of China; State Key Laboratory of Microbial Metabolism, Joint International Research Laboratory of Metabolic & Developmental Sciences, School of Life Sciences & Biotechnology, Shanghai Jiao Tong University, 800 Dongchuan Road, Shanghai 200240, People's Republic of China; State Key Laboratory of Microbial Metabolism, Joint International Research Laboratory of Metabolic & Developmental Sciences, School of Life Sciences & Biotechnology, Shanghai Jiao Tong University, 800 Dongchuan Road, Shanghai 200240, People's Republic of China; Shanghai Public Health Clinical Center, State Key Laboratory of Genetic Engineering, Collaborative Innovation Center of Genetics and Development, Department of Physiology and Biophysics, School of Life Sciences, Fudan University, Shanghai 200433, People's Republic of China; State Key Laboratory of Microbial Metabolism, Joint International Research Laboratory of Metabolic & Developmental Sciences, School of Life Sciences & Biotechnology, Shanghai Jiao Tong University, 800 Dongchuan Road, Shanghai 200240, People's Republic of China; State Key Laboratory of Microbial Metabolism, Joint International Research Laboratory of Metabolic & Developmental Sciences, School of Life Sciences & Biotechnology, Shanghai Jiao Tong University, 800 Dongchuan Road, Shanghai 200240, People's Republic of China

## Abstract

The sulfur atom of phosphorothioated DNA (PT-DNA) is coordinated by a surface cavity in the conserved sulfur-binding domain (SBD) of type IV restriction enzymes. However, some SBDs cannot recognize the sulfur atom in some sequence contexts. To illustrate the structural determinants for sequence specificity, we resolved the structure of SBD_Spr_, from endonuclease SprMcrA, in complex with DNA of G_PS_GCC, G_PS_ATC and G_PS_AAC contexts. Structural and computational analyses explained why it binds the above PT-DNAs with an affinity in a decreasing order. The structural analysis of SBD_Spr_–G_PS_GCC and SBD_Sco_–G_PS_GCC, the latter only recognizes DNA of G_PS_GCC, revealed that a positively charged loop above the sulfur-coordination cavity electrostatically interacts with the neighboring DNA phosphate linkage. The structural analysis indicated that the DNA–protein hydrogen bonding pattern and weak non-bonded interaction played important roles in sequence specificity of SBD protein. Exchanges of the positively-charged amino acid residues with the negatively-charged residues in the loop would enable SBD_Sco_ to extend recognization for more PT-DNA sequences, implying that type IV endonucleases can be engineered to recognize PT-DNA in novel target sequences.

## INTRODUCTION

Bacterial DNA phosphorothioate (PT) modification involves the replacement of the *R*_P_ non-bridging oxygen of a given phosphodiester bond with sulfur by Dnd proteins (1,2). Natural PT modifications dynamically occur to both DNA strands at the consensus sequences of G_PS_GCC (_PS_ denotes PT link) in *Streptomyces lividans 66*, G_PS_AAC/ G_PS_TTC in *Escherichia coli* B7A and *Salmonella enterica* 87, and G_PS_ATC in *Bermanella marisrubri* RED65, or to a single strand at C_PS_CA sites in *Vibrio cyclitrophicus* FF75 (3,4). *dnd* gene clusters governing PT modification are present in more than 1,300 bacterial and archaeal species (5,6). PT modification has been implicated in conferring resistance to oxidation to the host bacteria (7,8), influencing the global transcriptional response (9), and participating in restriction-modification systems in bacteria (10).

DNA modifications, primarily base methylation, participate in DNA replication and gene regulation through interactions with different nucleic acid binding proteins, also known as ‘readers’ (11), which transmit methylation information to other systems. For example, 5mCpG, the major eukaryotic methylated dinucleotide, is recognized by the methyl-GpG-binding (MBD) domain and the SET and RING finger-associated (SRA) domain, the prevalent 5mC reader in three life kingdoms (12,13). The SRA domain is often fused to other domain(s) that function in versatile cellular processes related to 5mC metabolism, or fused with a nuclease motif to cleave DNA in a modification-dependent way (14–18). Therefore, studies of the recognition mechanism of DNA modification by these readers are important to understand the flow of epigenetic information.

We recently identified a new type of reader in the *Streptomyces coelicolor* type IV restriction enzyme ScoMcrA, which specifically recognizes and cleaves PT-modified DNA (19), and this reader is a sulfur-binding domain (SBD) (20,21). PT-dependence is dictated by SBDs, whose carboxyl termini almost exclusively contain HNH nuclease motifs (21).

The complex structure of SBD_Sco_ bound to the PT-DNA sequence 5′-CCG_PS_GCCGG-3′ was determined [Protein Data Bank (PDB) accession number: 5ZMO] (20). Discrimination of sulfur from oxygen by the SBD was achieved by concurrent interactions with the sulfur atom, the non-bridging oxygen of neighboring phosphates, as well as with the base pairs surrounding the PT linkage, in a manner generally similar to the recognition of 5mC or 5hmC by SRA. SBD_Sco_ only contacted the PT linkage on one DNA strand even though both strands were phosphorothioated. Three phosphates in the vicinity of the central PT link were wrapped in a cleft edged by two positively-charged patches on the surface of SBD_Sco_, where the *R*_P_ sulfur was firmly coordinated by a hydrophobic concave pointing to the bottom of this cleft (20). SBD_Sco_ only binds to PT-DNA in the sequence G_PS_GCC and not to the other four natural PT modification sequences, in sharp contrast to that the SRA domain has high flexibility in target sequence selection (22). In the complex structure of SBD_Sco_–G_PS_GCC, four residues on a ‘base-contacting’ loop formed seven hydrogen bonds with the three base pairs across the PT link. Single mutation of individual residues lowered the binding affinity to G_PS_GCC by varying amounts, ranging from 20% to 80% (20). By contrast, the SBD of the PT-dependent restriction endonuclease (REase) SprMcrA from *Streptomyces pristinaespiralis* has a relatively relaxed sequence specificity, targeting G_PS_GCC, G_PS_AAC and G_PS_ATC, but not G_PS_TTC or C_PS_CA (21).

To understand the reasons underlying the differences in sequence specificity among SBDs, we crystalized SBD_Spr_ with G_PS_GCC, G_PS_AAC and G_PS_ATC. Comparative structural analysis revealed that a surface patch on two SBDs possesses a reverse charge, which exerts repelling and attracting strength on DNA by SBD_Sco_ and SBD_Spr_, respectively. Mutation of E156R/D157R in this patch from SBD_Sco_ conferred the mutant domain with the ability to bind G_PS_AAC and G_PS_ATC. Additionally, we provide evidence for why both SBDs showed a higher affinity for G_PS_GCC than for the other DNA sequences. This study reports that variation in DNA binding affinity constitutes a key determinant of the sequence specificity for SBDs and provides new insights into approaches for engineering the specificity of modification-dependent REases by altering their contacts with DNA phosphates other than the nucleotide bases.

## MATERIALS AND METHODS

### Construction of protein expression vector and site-directed mutagenesis

DNA fragments encoding wild-type SBD_Spr_ and SBD_Sco_ were cloned into the pET28a vector (Novagen), with N-terminal 6xHis tags. His-tagged SBD_Spr_ and SBD_Sco_ mutant variants were constructed by the whole-plasmid PCR and *DpnI* digestion method (23). The *Escherichia coli* strain DH10b was used as a transformation host. The mutations were confirmed by DNA sequencing of the entire gene. Primers used for plasmid construction were listed in Supplementary Table S1.

### Preparation and purification of stereospecific PT-DNA

The PT-DNA oligonucleotides were chemically synthesized and PAGE-purified. The concentration of oligonucleotides was determined by spectrophotometric measurement on a NanoDrop 2000 spectrophotometer (Thermo), and double-stranded DNA was prepared by mixing equimolar concentrations of complementary oligonucleotides, followed by heating to 95°C for 2 min and gradual cooling. The *R*_P_ and *S*_P_ stereoisomers of double-stranded PT-DNA were separated by anion exchange HPLC with a DNAPac PA-100 analytical column (Thermo) on an Agilent 1260 Infinity Series system at a flow rate of 1 ml/min with the following parameters (column at room temperature; solvent A, 10 mM Tris–HCl, pH 8.0; solvent B, 10 mM Tris–HCl, pH 8.0, 1 M NaCl; gradient, 10% B to 70% B over 40 min; detection by UV absorbance at 260 nm). The eluent was desalted with a Copure C18 column (Biocomma), dried on an RVC 2–25 rotational vacuum concentrator (Martin Christ), and dissolved with distilled deionized water.

### Protein expression and purification

Proteins were expressed in the *Escherichia coli* strain BL21(DE3) at 16°C; a 10-ml culture grown overnight from a single colony was inoculated into 1 l of Luria Broth medium supplied with 50 ug/ml kanamycin. The culture was incubated at 37°C to an OD_600_ of 0.6–0.8 and induced by the addition of 0.2 mM isopropyl-d-1-thiogalactopyranoside (IPTG) for another 20 h at 16°C. The cells were harvested and resuspended in 20 ml binding buffer (20 mM MES, pH 6.8, 20 mM imidazole, and 300 mM NaCl) and lysed by sonication in an ice bath. After centrifugation at 16 000 g for 60 min at 4°C, the supernatant was applied to 2 ml Ni-NTA column (GE Healthcare) pre-equilibrated with binding buffer. The Ni-NTA column was eluted with 10 ml of elution buffer (20 mM MES, pH 6.8, 300 mM imidazole and 300 mM NaCl) after washing. The His_6_-tagged protein products were purified with a HiTrap Heparin HP affinity chromatography column (GE Healthcare), and a Superdex200 10/300 GL gel filtration chromatography column (GE Healthcare) equilibrated with 10 mM Tris–HCl (pH 8.0), 100 mM NaCl and 1 mM DTT, using an AKTA FPLC system (GE Healthcare). The peak fractions were combined and concentrated to 10 mg/ml. Purified proteins were visualized by Coomassie-stained 15% SDS-PAGE analysis, and protein concentration was determined using a Bradford Protein Assay Kit (Bio-Rad).

### Crystallization, data collection and structure determination

Crystals for SBD_Spr_ in complex with the *R*_P_ form of the 8-bp hemi-PT DNA oligonucleotide 5′-GGCG_PS_GCCC-3′ were grown at 14°C using the sitting-drop vapor-diffusion method in 48-well plates (24). Typically, 1 μl of reservoir solution was mixed with 1 μl of protein–DNA solution and equilibrated against 80 μl of reservoir solution. After optimization and macroseeding efforts, diffracting crystals of SBD_Spr_–G_PS_GCC were obtained from a buffer of 0.01 M magnesium acetate tetrahydrate, 0.05 M sodium cacodylate trihydrate pH 6.5, and 1.3 M lithium sulfate monohydrate. Crystal diffraction datasets at a resolution of 2.06 Å for the SBD_Spr_–G_PS_GCC complex were collected at the BL19U1 beamline at the National Center for Protein Science Shanghai and processed using HKL2000 (25). The crystal belonged to space group *P*6_1_22, and contained three molecules of SBD_Spr_ in complex with three molecules of PT-DNA in each asymmetric unit. The crystal structure was determined by the molecular replacement method with the Phaser program (26), using the structure of SBD_Spr_–G_PS_AAC as the searching model. The structure of the SBD_Spr_–G_PS_GCC complex was refined and rebuilt using Coot (27) and Refmac (28).

The co-crystal of SBD_Spr_ with 8-bp oligos with G_PS_AAC sequence was not successfully obtained. Crystals for SBD_Spr_ in complex with the *R*_P_ form of the 10-bp hemi-PT DNA oligonucleotide 5′-GGCG_PS_AACGTG-3′ were grown and obtained at 14°C with the reservoir solution containing 0.1 M BIS-Tris pH 5.5, 0.15 M ammonium acetate, and 25% PEG 3350. The SBD_Spr_–G_PS_AAC complex crystals belonged to the P1 space group, with two molecules of SBD_Spr_ and two molecules of G_PS_AAC–DNA; the structure of the complex was determined to 2.42 Å by the molecular replacement method with the phenix.rosetta_refine program (29), using the SBD domain of the ScoMcrA structure (PDB code: 5ZMO) as the searching model. The structure of the SBD_Spr_-G_PS_AAC complex was refined and rebuilt using Coot and Phenix.refine.

Crystals for SBD_Spr_ in complex with the *R*_P_ form of the 8-bp hemi-PT DNA oligonucleotide 5′-GATG_PS_ATCC-3′ were grown at 14°C with the reservoir solution containing 0.1 M Tris–HCl pH 8.5 and 4.5% PEG 8000. The SBD_Spr_–G_PS_ATC complex crystals belonged to the *C*222_1_ space group, with two molecules of SBD_Spr_ and two molecules of G_PS_ATC–DNA in the asymmetric unit; the structure of this complex was determined to 3.3 Å by the molecular replacement method with the Phaser program (30), using the structure of SBD_Spr_–G_PS_AAC as the searching model. The structure of the SBD_Spr_–G_PS_ATC complex was refined and rebuilt using Coot, Refmac and Phenix.refine.

The data collection statistics and the refinement statistics for the SBD_spr_–G_PS_GCC, SBD_Spr_–G_PS_AAC and SBD_Spr_–G_PS_ATC complexes are summarized in Supplementary Table S2.

### Electrophoretic mobility shift assay (EMSA)

Each EMSA reaction contained 6 pmol DNA and protein at a concentration 4-fold higher than the DNA concentration (molar ratio) in 10μl binding buffer (20 mM Tris–HCl, pH 8.0, 100 mM NaCl and 5% glycerol). After incubation at room temperature for 5 minutes, the reaction mixtures were loaded onto 12% non-denaturing polyacrylamide gels (acrylamide:bisacrylamide ratio of 79:1, w/w) and electrophoresed in 0.5× TBE buffer at 15 mA for 30 min. Ten bp-oligonucleotides used for EMSA assay were listed in Supplementary Table S1.

### Fluorescence polarization assay for analysis of DNA binding

5′-FAM-labeled hemi-PT-DNA, labeled on one strand only, was synthesized and purified (Supplementary Table S1). Protein solutions were diluted serially using 2-fold dilutions (5 μM starting concentration, 16–20 dilutions) and mixed with a 5 nM final concentration of DNA probe in a Corning 3575 plate, using binding buffer of 20 mM Tris–HCl pH 8.0, 5% glycerol, 50 mM NaCl and 1 mM DTT. The mixture was incubated for 10 min at room temperature, and fluorescence polarization was measured at room temperature on a SpectraMax i3x (Molecular Devices) using 485/20 nm and 528/20 nm filters for emission and excitation, respectively. The dissociation constants (*K*_D_) were calculated by fitting the experimental data (from two experimental replicates) to the following equation using GraphPad Prism software (version 6.0): [mP] = [maximum mP][C] /(*K*_D_ + [C]) + [baseline mP], and then the curve was replotted using percent saturation calculated as ([mP] – [baseline mP])/([maximum mP] – [baseline mP]), where mP is millipolarization and [C] is protein concentration. The binding experiments were performed under the same laboratory conditions.

### Transformation efficiency assay

The pACYCDuet™-1 vector (PT^−^) and its derivative (PT^+^) carrying the *dnd* gene cluster from *Salmonella enterica* serovar Cerro 87 were introduced to *E. coli* BL21(DE3), and competent cells of the resulting strains were prepared using the standard calcium chloride protocol. Transformation frequency was determined by introducing 100 ng pET28a derivatives carrying *scoMcrA* or its mutant variants to the competent cells. The number of *E. coli* colonies in each experiment was determined by serial dilutions. Each experiment was repeated three times and the mean value of the transformation frequency was reported.

### All-atom molecular dynamics simulation

The co-crystal structure of the SBD domain of ScoMcrA and the natural PT-DNA fragments (G_PS_GCC) was used as a starting model to build up the nucleotide-mutant models of G_PS_ATC and G_PS_AAC, and the protein-mutant models of E156R and E156R/D157R, with the modeling software package of Molecular Operating Environment v2018 (31). All the molecular dynamics simulations (MDs) were performed with the AMBER 16 software (30). For protein and DNA parts, Amber ff14SB and OL15 force field were used, respectively (32,33). The phosphorothioate force field employed these parameters developed by Mukherjee and Bhattacharyya *et al.* (34). The PROPKA algorithm determined the protonation of the SBD–DNA complex on the PDB2PQR web server (35). The protein-DNA complexes were then solvated within a cubic box and the TIP3P water model (36), in which the minimum distances between any protein atom and edges of the water box was set to be 12 Å. The systems were neutralized by adding appropriate numbers of Na^+^ and Cl^−^ ions. Long-range electrostatic interactions were calculated with the Particle-Mesh-Ewald (PME) method (37), and van der Waals interactions were truncated within 12 Å. The time interval was set as 2 fs, and the SHAKE (38) algorithm was used to constrain the bonds-connecting hydrogen atoms. The entire system was first minimized and heated up to 298 K before the production process. The CPPTRAJ tool implemented in the AMBER 16 software package was used for trajectory analyses, such as the popular root-mean square deviation (RMSD) and cluster analysis. Solvent accessible surface area (SASA) is a parameter that measures the fraction of the protein surface interacting with the solvent molecules. The term corresponding to the SASA was calculated through BIOVIA Discovery Studio (39).

### Binding Free Energy Calculation

The binding free energy between PT-DNA and SBD was calculated by the MM/GBSA approach (40), using the following equations,

$$\begin{equation*}\Delta {G_{{\rm{bind}}}} = {G_{{\rm{complex}}}}{\rm{\ }} - ({G_{{\rm{PT}} - {\rm{DNA}}}} + {G_{{\rm{Protein}}}}),\end{equation*}$$

$$\begin{equation*}\Delta {G_{{\rm{bind}}}} = {\rm{\ }}\Delta H - T \cdot \Delta S \approx \Delta {E_{{\rm{MM}}}} + \Delta {G_{{\rm{solv}}}} - T\Delta S,\end{equation*}$$

$$\begin{equation*}\Delta {E_{{\rm{MM}}}} = \Delta {E_{{\rm{int}}}} + \Delta {E_{{\rm{vdW}}}} + \Delta {E_{{\rm{ele}}}},\end{equation*}$$

$$\begin{equation*}\Delta {G_{{\rm{solv}}}} = \Delta {G_{{\rm{GB}}}}{\rm{\ }} + \Delta {G_{{\rm{SA}}}},\end{equation*}$$

where Δ*E*_int_ is neglectable with the single-trajectory strategy. The nonpolar part of the solvation free energy (Δ*G*_SA_) was calculated with the solvent-accessible surface area (SASA) through the LCPO algorithm (41), by using Δ*G*_SA_ = *γ* ⋅ SASA + β (the surface tension constants γ and β were set to 0.0072 and 0, respectively). The polar part of the solvation energy (Δ*G*_GB_) was estimated using the Generalized Born (GB) model proposed by Onufriev *et al.* (GB^OBC1^, igb = 2) (42). The Δ*E*_vdW_, Δ*E*ele, Δ*G*_GB_ and Δ*G*_SA_ terms were computed based on the 500 snapshots extracted from the last 20 ns MD trajectories. Each trajectory was calculated individually, and then all energies were analyzed statistically.

### Non-covalent interaction (NCI) analysis

The independent gradient model (IGM) (43) and reduced density gradient (RDG) (44) analyses were carried out using Multiwfn 3.6 program (45). Molecular plots were visualized with the VMD 1.9.3 program (46).

The IGM analysis depends on the topological characteristics of the electron density, ρ. The IGM descriptor δg^inter^ is calculated as the difference between the first derivatives of electron density of the whole system and the fragments:

$$\begin{equation*}\ {\rm{\delta g}}{\left( {\rm{r}} \right)^{inter}} = \left| {\nabla {\rho ^{{\rm{IGM}},{\rm{inter}}}}} \right|\ - \left| {\nabla \rho } \right|\end{equation*}$$

δg^inter^ > 0 indicates the presence of weak interactions and its magnitude denotes the interacting intensity.

The non-covalent interaction RDG method is an alternative method to reveal weak interlayer interactions (44), with a dimensionless form of electron density gradient norm function:

$$\begin{equation*}{\rm{RDG }}\left( {{r}} \right) = \frac{1}{{2{{\left( {3{\pi ^2}} \right)}^{1/3}}}}\ \frac{{\left| {\nabla \rho \left( r \right)} \right|}}{{\rho {{\left( r \right)}^{4/3}}}}\end{equation*}$$

The sign of the second eigenvalue of the electron density Hessian matrix, sign(λ_2_), was used in the RDG analyses to judge the attractive and repulsive interaction, that is, corresponding to negative and positive values of sign(λ_2_) ρ, respectively.

## RESULTS

### Affinity of SBD_Spr_ for PT-DNA of varied sequence contexts

The sulfur modification-dependent REases SprMcrA and ScoMcrA use an SBD to recognize the DNA backbone phosphorothioate link of the *R*_P_ stereoisomer, which is adopted by the naturally occurring PT modifications in five DNA core sequence contexts (Table 1) in prokaryotes. ScoMcrA only recognizes G_PS_GCC, whereas SprMcrA binds and shifts DNA of the sequences G_PS_GCC, G_PS_ATC and G_PS_AAC in EMSAs (21). To compare the affinity of SBD_Spr_ (aa 1–165 of SprMcrA) for PT-DNA of the five natural PT sequence contexts, a set of hemi-modified DNA duplexes, which differed from each other in the core sequence bearing the PT link in either the *S*_P_ or *R*_P_ configuration (Table 1) were assayed (Supplementary Figure S1). In agreement with the reported EMSA results (21), no *S*_P_ PT-DNA nor *R*_P_ PT-DNA in sequences of G_PS_TTC or C_PS_CA could be recognized by SBD_Spr_, indicating that recognition of the PT link is coupled with interactions with surrounding nucleobase or phosphate groups. SBD_Spr_ showed the highest binding affinity for G_PS_GCC, with a dissociation constant value (*K*_D_) of 5.55 nM, followed by a *K*_D_ of 38.33 nM for G_PS_ATC and 94.67 nM for G_PS_AAC (Table 1). By comparison, SBD_Sco_ showed a *K*_D_ of 102 nM for G_PS_GCC (Table 2), 18.5-fold weaker binding than SBD_Spr_ had to the same DNA duplex.

**Table 1.** Affinity of SBD_Spr_ for PT-DNA of different core sequences

PT-DNA		*K*_D_ (nM)
G_PS_GCC	*R*_P_	5.6 ± 0.9
	*S*_P_	–*
G_PS_ATC	*R*_P_	38 ± 8
	*S*_P_	–
G_PS_AAC	*R*_P_	95 ± 25
	*S*_P_	–
G_PS_TTC	*R*_P_	–
	*S*_P_	–
C_PS_CA	*R*_P_	–
	*S*_P_	–

*The binding affinity of SBD_Spr_ to substrate DNA was too weak, making the *K*_D_ value too large to be determined.

**Table 2.** *K*_D_ (nM) value of SBD_Sco_ and SBD_Sco_ mutants for PT-DNA of different core sequences

	Hemi PT-DNA duplex		
	G_PS_GCC	G_PS_ATC	G_PS_AAC
SBD_sco_ (wild type)	102 ± 12*	1091 ± 58	567 ± 37
E156D	112 ± 12	1322 ± 99	846 ± 74
E156Q	137 ± 18	1015 ± 49	618 ± 43
E156L	110 ± 12	791 ± 42	499 ± 37
E156K	130 ± 11	370 ± 14	297 ± 19
E156R	133 ± 21	346 ± 17	267 ± 15
E156R/D157R	123 ± 8	192 ± 12	183 ± 9

* *K*_D_ (dissociation constant, nM).

### Structure of SBD_Spr_ complexes

**Figure 1.**
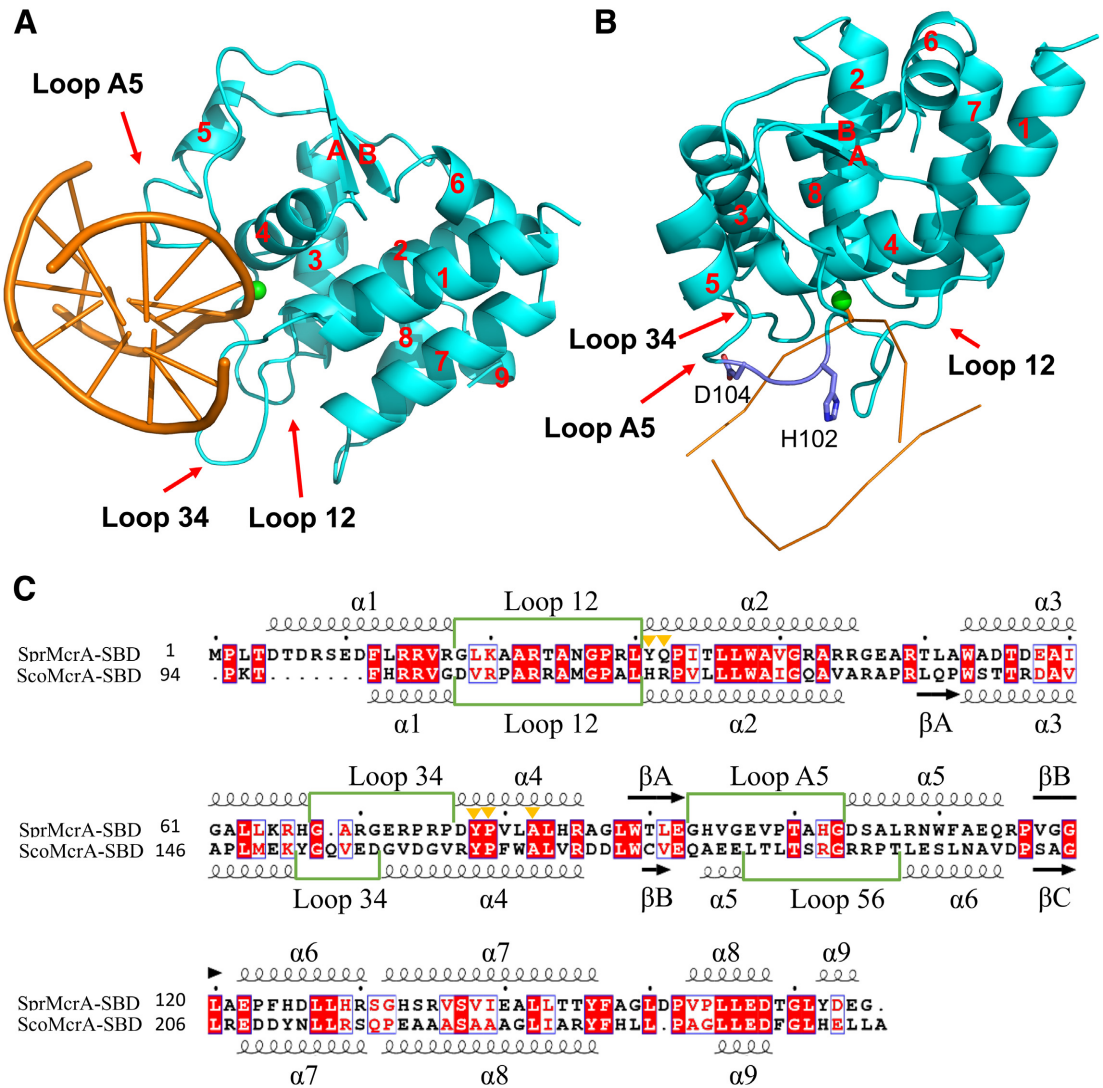
SBD of the PT-dependent restriction endonucleases SprMcrA and ScoMcrA. (**A**, **B**) Two views of the SBD of SprMcrA (SBD_Spr_) binding PT-DNA specifically. Loop 12, loop 34 and loop A5, which interact with DNA, are indicated by red arrows. The side chains of His102 and Asp104 (colored in purple) are located in the DNA major groove. The green ball in DNA molecule denoted the sulfur atom. (**C**) Sequence alignment of the SBD of SprMcrA (SBD_Spr_) and ScoMcrA (SBD_Sco_). The conserved residues forming the sulfur atom-binding pocket are marked with yellow triangles. Secondary structure elements of SBD_Spr_ and SBD_Sco_ are numbered according to crystal structures (PDB code: 7CC9 and 5ZMO). The loops that interact with DNA are marked by green boxes. Alignment was generated with ESPript.

**Figure 2.**
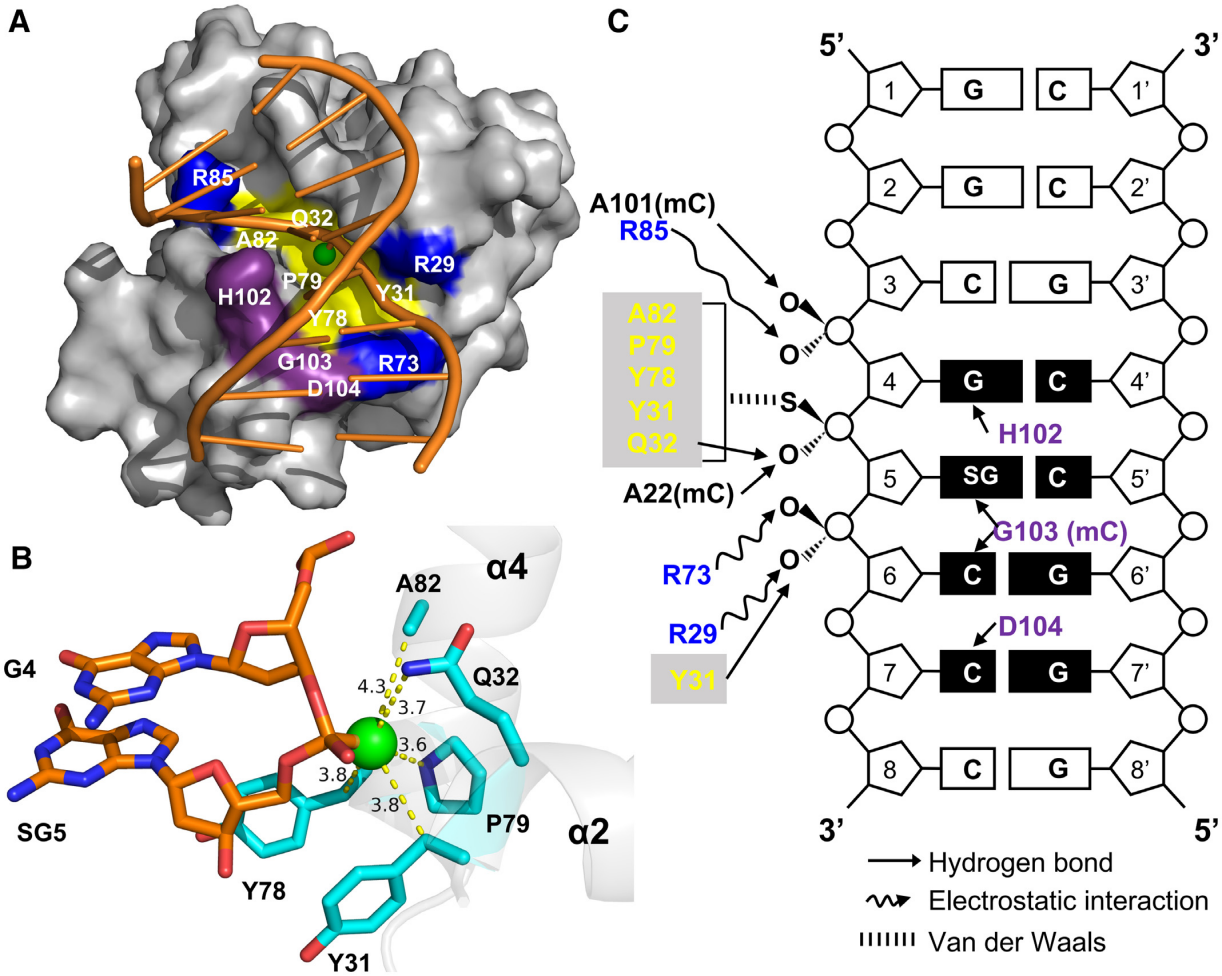
Details of the SBD_Spr_–DNA interactions. (**A**) The SBD_Spr_ binds specifically to G_PS_GCC. Residues that interact with DNA are colored as follows: Tyr31, Gln32, Tyr78, Pro79 and Ala82, which form the sulfur atom binding pocket, are in yellow; His102, Gly103 and Asp104, which recognize DNA bases, are in purple; and Arg29, Arg73 and Arg85, which interact with phosphates through electrostatic interactions, are in blue. (**B**) Sulfur atom-binding pocket on SBD_Spr_ formed by Tyr31, Gln32, Tyr78, Pro79 and Ala82. (**C**) Schematic summary of the interactions between SBD_Spr_ and PT-DNA, mC represented the main chain of amino acid.

**Figure 3.**
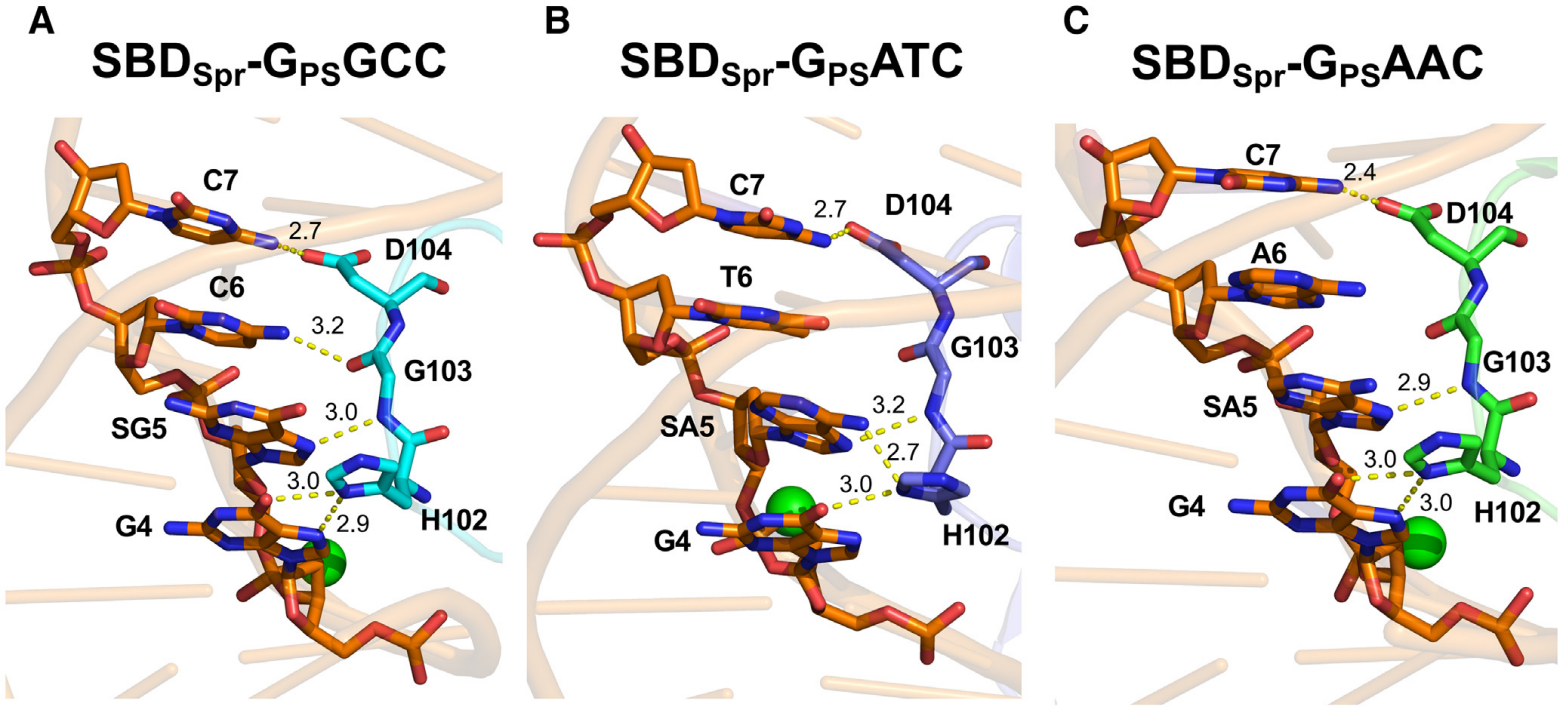
Comparison of HGD motif of SBD_Spr_ interaction with three DNA core sequences of (**A**) G_PS_GCC, (**B**) G_PS_ATC and (**C**) G_PS_AAC. SG5 and SA5 represents the nucleoside G5 and A5 with PT modification. The base contact motif HGD of SBD_Spr_ in the three complexes was shown in cyan, purple and green, respectively.

To determine why SBD_Spr_ showed varying affinity for G_PS_GCC, G_PS_ATC and G_PS_AAC, as well as a much higher affinity for G_PS_GCC, we determined the crystal structures of SBD_Spr_ in the presence of three hemi-PT-DNA oligonucleotides with the G_PS_GCC, G_PS_ATC and G_PS_AAC core sequences (PDB codes 7CC9, 7CCJ and 7CCD, respectively; Supplementary Table S2). The overall structures for the three complexes were similar with in the terms of sulfur coordination (Supplementary Figure S2) except for different base interaction (See below). To simplify the description of the SBD_Spr_ structure and facilitate comparative analysis with SBD_Sco_-G_PS_GCC, we here depict the details on one of the complexes, SBD_Spr_–G_PS_GCC as an example. The structure was determined by molecular replacement and refined to a resolution of 2.06 Å. The crystallographic asymmetric unit contained three protein molecules, with each of them associated with one molecule of hemi-PT-DNA (Supplementary Figure S3A). The three SBD molecules and their respective DNA molecules in a crystallographic unit were well aligned with each other (the value of root mean square error is less than 0.140 Å over 165 Cα atoms) (Supplementary Figure S3D). SBD_Spr_ comprised nine helices (α1–α9) and two β-sheets (βA and βB) (Figure 1A–C). SBD_Spr_ clearly did not make any contact with the DNA strand without the PT linkage. The sulfur atom from the DNA helical edge was positioned outward into the central bottom of a hydrophobic cavity that was formed by the side chains of five amino acid residues from the separated helices 2 and 4 (Figure 2A and B). The PT-DNA binds within a basic groove on the wedge-like surface of the SBD protein, leading to the mortise-and-tenon-like interactions (Figure 2A). The sulfur atom of the PT linkage was inserted into a cavity which formed by Y31, Q32, Y78, P79 and A82 through Van der Waals interactions; the phosphate groups flanking the PT linkage formed electrostatic bonds with R29, R73 and R85, as well as hydrogen bonds with Y31 and A101 (Figure 2C). As predicted, a single mutation within the five residues diminished the DNA binding affinity to varied extents, particularly with either of the two Y→A mutations, which almost abolished DNA binding affinity (Supplementary Figure S4, Table S3). In addition to interactions with the sulfur atom and phosphate backbone, the H102–G103–D104 motif of loop A5 inserted into the PT-DNA major groove to form five hydrogen bonds (H-bond) with bases of G_PS_GCC core sequence (Figures 1B, 2C and 3).

### Comparison of the structure of SBD_Spr_ and SBD_Sco_ complexed with G_PS_GCC

Generally, the overall structure of the SBD_Spr_ monomer, including the sulfur-binding cavity, was similar to SBD_Sco_ except for three flexible loops that swayed differently (Supplementary Figure S5). Among them, loop 12 made no contact with DNA substrates in either structure. Loop 34 was three amino acids longer in SBD_Spr_ than in SBD_Sco_ and made contacts with the DNA phosphate backbone. The location of loop A5 in SBD_Spr_ was shifted by four amino acids from the equivalent loop 56 in SBD_Sco_ (Figure 1C); however, both of these loops were inserted into the major groove of the DNA substrates and contacted the DNA bases (Figure 1A, B). The sequence and spatial arrangement of these residues involved in sulfur coordination were well aligned with the equivalent residues of SBD_Sco_, but differed slightly by the presence of an additional electrostatic bond with the guanidine group of R117 in SBD_Sco_ (Supplementary Figure S6). The *S*_P_ oxygen symmetric to sulfur was stabilized by two hydrogen bonds with Q32 and A22 in SBD_Spr_ whereas it only bonded to the imino group of R117 in SBD_Sco_. The Q32A mutation in SBD_Spr_ caused an ∼50-fold decrease in binding affinity, but the equivalent mutation of R117A or R117G in SBD_Sco_ completely abolished the affinity for PT-DNA, possibly due to the loss of three bonds between R117 and the sulfur and oxygen atoms.

Except for the electrostatic interaction between the phosphate group of G^4^ and R85, the remaining four bonds lack equivalents in the structure of the SBD_Sco_ complex (20). Therefore, the striking structural difference between the two complexes with respect to the interaction with the phosphate backbone lies in the lack of any interaction with the phosphate group of C^6^, immediately downstream of the phosphorothioate in SBD_Sco_. On the contrary, in SBD_spr_, Y31 also makes a hydrogen bond to the fifth DNA phosphate in addition to coordination with the sulfur atom (Figure 2C).

### Base contact by SBD_Spr_ determines the variation in binding affinity

As mentioned above, SBD_Spr_ displayed varied affinity to PT–DNA of different core sequences (Table 1). In three SBD_Spr_ co-crystal structures, the H102–G103–D104 motif all inserted into the DNA major groove to make contacts with bases, but the numbers of H-bonds were slightly different (Figure 3). In all structures, the ND1 atom of H102 formed H-bond with O6 atom of G^4^, and the OD2 atom of D104 bonded to the N4 atom of C^7^. It's worth noting that the ND1 atom of H102 also formed H-bond with N7 atom of G^4^ in G_PS_GCC and G_PS_AAC sequences, while this H-bond was not existing in SBD_Spr_-G_PS_ATC complex. When the central SG^5^C^6^ are changed to SA^5^A^6^ or SA^5^T^6^, the H-bonds patterns formed by the central bases showed some differences in three complexes. The N atom in the main chain of G103 bonded to N7 atom of SG^5^ from G_PS_GCC sequence and N7 atom of SA^5^ from G_PS_ATC and G_PS_AAC sequences. The carbonyl O atom of G103 formed an additional H-bond with N4 atom of base C^6^ in SBD_Spr_-G_PS_GCC complex. SA^5^ showed a significant deflection in G_PS_ATC sequence, compared with G_PS_GCC and G_PS_AAC sequences, leading to formation of another H-bond between the ND1 atom of H102 with the N6 atom of SA^5^. In conclusion, HGD motif formed five H-bonds in SBD_Spr_–G_PS_GCC complex while four H-bonds in SBD_Spr_–G_PS_ATC and SBD_Spr_–G_PS_AAC complexes, which explained why SBD_Spr_ showed a highest affinity for G_PS_GCC.

The base recognition pattern by HGD motif in complexes of SBD_Spr_–G_PS_AAC and SBD_Spr_–G_PS_ATC is different (Figure 3). What's more, close comparison of two structures revealed that the binding of the G_PS_AAC released the methyl group of T6′ on the complementary strand from the binding site, and converts the weak non-bonded interaction to unfavored thymine methyl-solvent accessibility (Supplementary Figure S7). In the case of G_PS_ATC, the percent solvent accessibility of T6 methyl group was calculated to be only 10–15, corresponding to fully buried with the SBD-DNA interface. The multiple C–H…O contacts between the methyl and Y78 were believed to be attractive for G_PS_ATC (47). In the case of G_PS_AAC, the T6′ methyl group was fully exposed to solvent and lack of specific interaction with SBD protein. Difference in the weak non-bonded interaction between G_PS_ATC and G_PS_AAC results in a lower *K*_D_ values for G_PS_ATC than G_PS_AAC bound by SBD_Spr_.

To evaluate the contribution of these interactions with the bases to the DNA binding affinity, the three aa residues HGD were independently mutated, and the binding affinity of the resulting mutated proteins to hemi-PT-DNA of 5′-GGCG_PS_GCCC-3′ was measured by fluorescence polarity (Supplementary Table S3). The H102A and D104A mutants showed a 370-fold and 25-fold decrease, respectively, in binding affinity compared with wild-type protein, demonstrating that base contact constitutes an important component of the total affinity for PT-DNA by ensuring the formation of a stable DNA/protein complex. Unexpectedly, the G103A mutation almost abolished the affinity for PT-DNA as evidenced by the strikingly increased *K*_D_ value of >9000 nM (Supplementary Table S3). The G103A mutation introduced an additional C-C side chain, which increased the main chain rigidity and affecting the hydrogen bonding network of base G^5^ and C^6^, leading to drastic decrease in binding affinity.

### Opposite interactions with PT-DNA by loop 34 of SBD_Spr_ and SBD_Sco_

**Figure 4.**
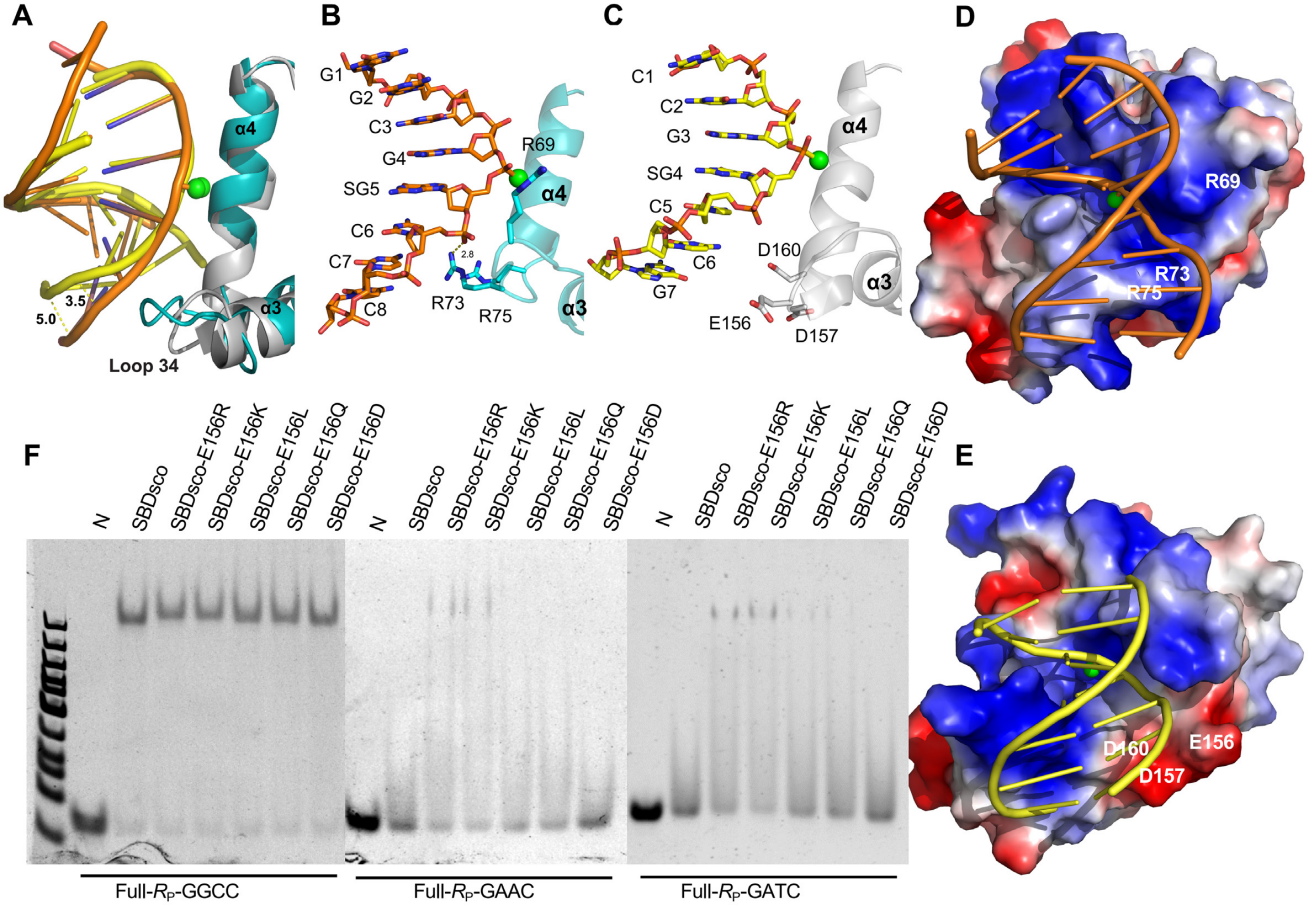
Comparison of SBD_Spr_ and SBD_Sco_ interactions with PT-DNA. (**A**) Superimposition of SBD_Spr_ loop34 (cyan) with DNA (orange) and SBD_Sco_ loop34 (grey) with DNA (yellow). Phosphorus atoms of the seventh and eighth bases in the PT-modified strand of SBD_Sco_-G_PS_GCC were extruded by 3.5 and 5.0 Å relative to those in the SBD_Spr_–G_PS_GCC. (**B**) Arg69, Arg73 and Arg75 of SBD_Spr_ loop 34 form a positive interface with the DNA. (**C**) Glu156, Asp157 and Asp160 of SBD_Sco_ loop 34 and helix 3 form a negatively charged surface area to DNA. (**D**, **E**) The surface charge of (D) SBD_Spr_ and (E) SBD_Sco_. The surface charge distribution at neutral pH is displayed with blue for positive, red for negative, and white for neutral. (**F**) Influence of mutations in E156 on the ability of SBD_Sco_ to bind G_PS_GCC, G_PS_ATC and G_PS_AAC in EMSAs. N, no protein added to the EMSA.

When the structures of SBD_Spr_–G_PS_GCC and SBD_Sco_–G_PS_GCC were compared, a striking DNA strand distortion at the two phosphodiester bonds proximal to the 3′ terminus of the PT-DNA strand was observed in the SBD_Sco_–G_PS_GCC complex. Phosphorus atoms of the seventh and eighth bases in the PT-modified strand were extruded by 3.5 and 5.0 Å relative to those in the SBD_Spr_–G_PS_GCC structure. (Figure 4A). Compared with SBD_Sco_, SBD_Spr_ possesses a longer loop 34, containing the three positively charged residues R69, R73, and R75, which constitute a local positive interface with DNA wherein R73 bonds to the phosphate group of C^6^ (Figure 4B, D). By contrast, the corresponding interface of the SBD_Sco_ loop34 features two tandem acidic residues, E156 and D157, and a spatially adjacent D160 (Figure 4C, E). These residues form a negatively charged surface area, which is repulsive towards the DNA phosphate backbone and may account for the distortion of the DNA double helix structure in the SBD_Sco_–G_PS_GCC complex.

### Ability of SBD_Sco_–E156R/D157R to bind PT-DNA of G_PS_AAC and G_PS_ATC

Given that SBD_Sco_ can only bind to G_PS_GCC, and that SBD_Spr_ has maximum affinity for G_PS_GCC, we hypothesized that the repulsive force exerted by the negative interface of SBD_Sco_ weakened its overall affinity for PT-DNA, leading to the failure to recognize G_PS_AAC or G_PS_ATC, although this repulsive force was not sufficient to disrupt the most stable complex formed with G_PS_GCC.

**Figure 5.**
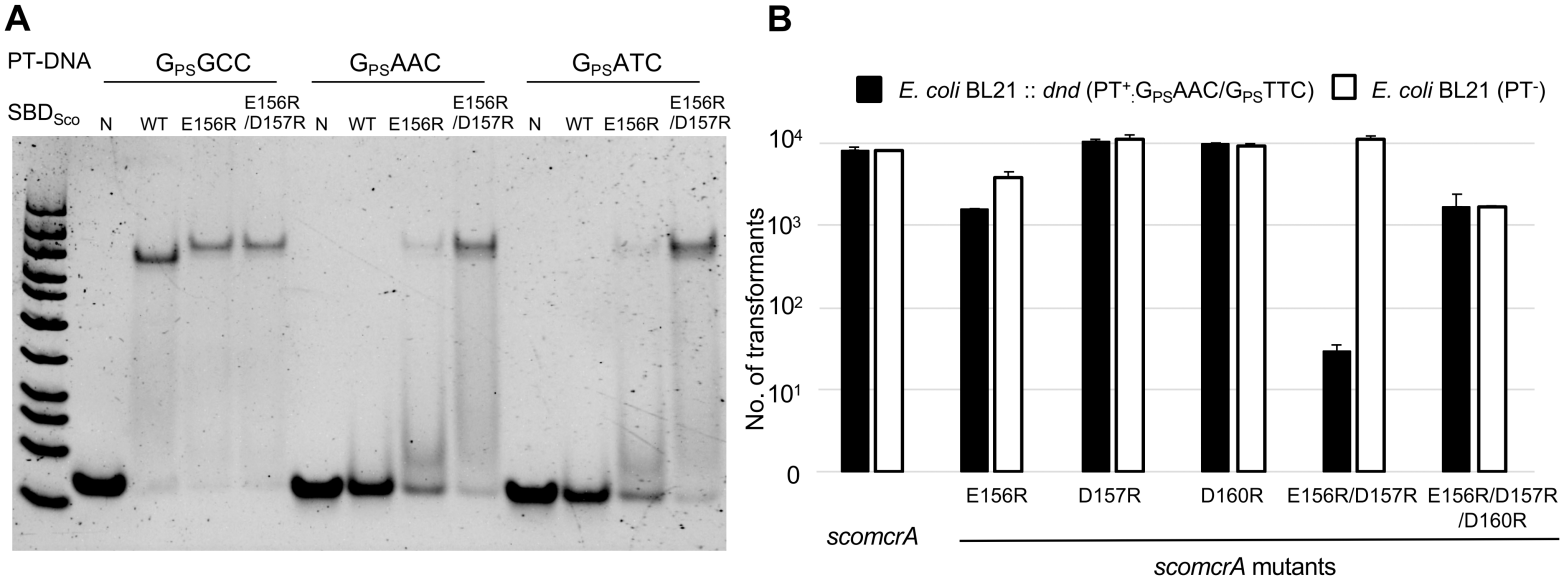
Sequence specificity of ScoMcrA mutants. (**A**) Ability of SBD_Sco_ mutants to bind G_PS_GCC, G_PS_ATC and G_PS_AAC in EMSAs. N, no protein added to the EMSA; WT, the wild-type SBD_Sco_; E156R, SBD_Sco_–E156R mutant; E156R/D157R, SBD_Sco_–E156R/D157R mutant. (**B**) Uptake efficiency of *scoMcrA* and its mutants by the PT^−^ host and PT^+^ host. The PT^+^ host contains the expression vector with the *dnd* gene cluster from *Salmonella enterica*, which encodes the ‘writer’ proteins for phosphorothioation of G_PS_AAC /G_PS_TTC. Transformation efficiency obtained with the *dnd* host (PT^+^) and the negative control host (PT^−^) is indicated by black bars and white bars, respectively.

To test this hypothesis, E156 of SBD_Sco_, structurally equivalent to R73 of SBD_Spr_, was mutated into basic (R and K), neutral (L and Q), and acidic (D) residues. Affinity quantification of each E156 mutant by fluorescence polarization assay showed that the mutants containing the R, K or L substitutions all showed an increased affinity for G_PS_AAC and G_PS_ATC relative to the wild-type protein. In particular, the E156R mutant displayed the most significant increases in DNA binding affinity for G_PS_ATC and G_PS_AAC by, respectively, ∼3-fold and ∼2-fold (Table 2, Figure 4F, Supplementary Figure S8). The double-mutation protein, E156R/D157R, showed further increases in DNA binding affinity for G_PS_ATC and G_PS_AAC in EMSA (Figure 5A), which were quantified to be ∼5.7-fold and ∼3-fold increases for G_PS_ATC and G_PS_AAC, respectively (Table 2). Unfortunately, we were unable to purify the triple-mutation protein, E156R/D157R/D160R, probably because the significant decrease in protein expression. However, mutations of the three acidic residues were constructed in the full-length ScoMcrA, and the *in vivo* nuclease activities of the mutants were analyzed by comparing the transformation efficiency of their coding DNA into a PT and non-PT *E. coli* host (Figure 5B). In agreement with the EMSA results for the SBD mutants, the uptake efficiency of *scoMcrA_E156R_* by the PT host was 2.5-fold less than by the non-PT host, while that of other two single-mutant genes had no significant difference in the PT and non-PT hosts. In parallel, the transformation efficiency of *scoMcrA_E156R/D157R_* was 500-fold less with PT *E. coli* than with non-PT *E. coli* (Figure 5B), implying that the double-mutation protein acquired restriction activity for G_PS_AAC DNA, but kept the ability to discriminate the unmodified DNA. However, the triple-mutant gene did not show distinctive transformation efficiency between the PT and non-PT *E. coli* hosts, but rather exhibited decreased efficiency with both hosts.

To further explore the key structural parameters that affect the binding of PT-DNA and protein, we performed multiple 100 ns MD simulations of the wild-type complex, as well as of the single mutation (E156R) and double mutation (E156R/D157R) complexes for SBD_Sco_–G_PS_ATC and SBD_Sco_–G_PS_AAC, which were built and mutagenized through Molecular Operating Environment. The most populated conformations sampled during our simulations that contain the interaction regions were chosen for RDG analysis. The calculated RDG isosurfaces with BGR color scales representing sign(λ_2_) ρ values are given in Supplementary Figure S9 for E156R and E156R/D157R in SBD_Sco_-G_PS_ATC and SBD_Sco_-G_PS_AAC. In E156R, two hydrogen bonds, N7_G_^7^⋅⋅⋅NH1_R156_ and O6_G_^7^⋅⋅⋅NH2_R156_, were strong. When we introduced a double mutation (E156R/D157R), two newly formed hydrogen bonds (HH12_R157_⋅⋅⋅O6_G_^7^ and HH22_R156_⋅⋅⋅O2P_C_^6^) were observed, similar to the case of E156R. Additionally, van der Waals interactions were also observed between the adjacent HH22_R157_ and O6_G_^7^ and N7_G_^7^, indicated by the green color of the RDG isosurfaces. The gain of G_PS_ATC and G_PS_AAC interactions with both R156 and R157 might be crucial to triggering the changes leading to the acquisition of the enhancement of non-covalent interaction. Replacement of E156 and D157 with arginine introduced hydrogen bond interactions as well as van der Waals interactions, which significantly strengthened the binding, resulting in a concerted interplay of interactions between the SBD and PT-DNA.

## DISCUSSION

Recognition of PT-DNA by the SBD of the type IV REase ScoMcrA is not only phosphorothioate-dependent but also DNA sequence-specific as this enzyme only recognizes PT-DNA of the G_PS_GCC core *in vivo* and *in vitro*, whereas it does not bind to the PT-DNA of other four core sequences found in prokaryotes. However, five SBD_Sco_ homologs, including SprMcrA, generally display a more relaxed sequence specificity in target DNA selection. Although both SBD_Spr_ and SBD_Sco_ clearly shifted the G_PS_GCC DNA duplex in EMSAs, affinity quantification showed that the former had an 18.2-fold higher affinity than the latter did, with SBD_Spr_ also showing a 28.7-fold and 6-fold higher affinity for G_PS_ATC and G_PS_AAC, respectively (Tables 1 and 2). These differences in binding affinity between the two SBD domains lead to a distinctive presence or absence of *in vivo* restriction activity. For example, ScoMcrA can restrict the uptake of *dnd* gene clusters generating the G_PS_GCC modification (20) but not those generating the GAAC/GTTC modifications (Figure 5B). In contrast, SprMcrA can block the establishment of PT modifications at G_PS_AAC/G_PS_TTC and G_PS_GCC sites as it showed an overall higher affinity for PT-DNA compared to ScoMcrA. Through comparative analysis, we attributed this difference in binding affinity to the reverse charge of loop 34 in both structures, which functions like a switch by changing between positive and negative electric charges in the different structures. Mutations of negatively charged amino acids into positively charged ones on loop 34 of SBD_Sco_ significantly enhanced the binding affinity for PT-DNA. For example, the E156R/D157R mutation conferred SBD_Sco_ with the ability to bind to G_PS_AAC and G_PS_ATC, and thus conferred ScoMcrA with *in vivo* restriction activity for *dnd* encoding G_PS_AAC/G_PS_TTC (Figure 5B). This structural switch offers us an opportunity to engineer a flexible or stringent sequence specificity for a given SBD.

In our study, the SBD_Sco_–E156R and SBD_Sco_–E156R/D157R mutants had significant increases in binding affinity for G_PS_ATC and G_PS_AAC when compared with the wild-type SBD_Sco_ (Table 2). The MD simulations showed that R156 and R157 participate in van der Waals interactions and hydrogen-bond interactions with C^6^ and G^7^ of PT-DNA with G_PS_ATC and G_PS_AAC core sequences, resulting in the higher binding affinity compared to the wild-type SBD_Sco_. However, the binding affinity of these two mutants for G_PS_GCC showed a slight decrease in comparison to the wild-type protein, in contrast to the increased affinities for G_PS_ATC and G_PS_AAC. The superposition of the mutants and wild type structures after MD simulations gave a root mean square deviation (RMSD) value of 0.636 Å by using backbone atoms (Cα), indicating the mutations do not lead to vastly structural changes in the MD simulations (Supplementary Figure S10). Next, the binding affinity of G_PS_GCC and SBD_Sco_ were carefully examined to understand the geometrical disturbance of E156R/D157R mutation with the MM/GBSA method (40). As shown in Supplementary Table S4, the ΔΔ*G*_binding_ value for PT-DNA binding SBD_Sco_–E156R/D157R was positive (0.9 kcal/mol), suggesting that the mutation slightly weakened the binding affinity, consistent with the experimental observation (Table 2). It is noticed that deformability of the DNA structure may contribute to the sequence specificity (48). As the conclusions of MD simulations, R156 and R157 are not directly involved in influencing binding interaction with C^6^ and G^7^ in G_PS_GCC sequence, however, they may affect the orientation of other residues that are involved in direct interaction with PT-DNA. We speculate that will lead to the twisting of DNA, which then results in an imperfect match of the hydrophobic pocket with the *R*_P_ sulfur atom, thus reducing the affinity. These interactions ultimately lead to an overall decrease in the binding affinity of mutant SBD_Sco_–E156R/D157R to G_PS_GCC. Interestingly, the SBD_Sco_–E156R/D157R mutant gained the ability to bind with the *S*_P_ stereoisomers of G_PS_GCC (Supplementary Figure S11) probably because the twisting of the G_PS_GCC strand by the E156R/D157R mutation positioned the sulfur of *S*_P_ within the sulfur-coordination cavity.

SBD homologs are widely represented in at least 1059 sequenced species from 14 phyla of bacteria (20). In addition to SBD_Sco_, four SBD homologs, including SBD_Spr_, displayed flexibility in the selection of substrate PT-DNA with different core sequences (20). It is notable that loop 34 is rich in basic amino acids in four of the SBDs (Supplementary Figure S12). With its acidic amino acids, loop 34 of SBD_Sco_ is unique among SBD homologs, which may be related to the unique domain composition of SBD-SRA-HNH for ScoMcrA. Multiple DNA recognition domains of ScoMcrA result in reduced ability to distinguish between modified and non-modified DNA substrates, in turn resulting in nonspecific cleavage activity. In order to maintain the specificity of cleavage activity and low toxicity, the distribution of positive charges on the surface may have become reduced in ScoMcrA during evolution. Consequently, ScoMcrA can only recognize and restrict G_PS_GCC, the most common core sequence of PT-DNA in *Streptomyces*, with flexibility lost in the selection of substrate PT-DNA with different core sequences. Overall, our study illustrates structural features that impact the recognition of PT-DNA by SBDs of type IV restriction enzymes.

## DATA AVAILABILITY

Atomic coordinates and structure factor for the sulfur-binding domain (SBD) from full-length *Streptomyces pristinaespiralis* endonuclease SprMcrA in complex with 5′-GGCG_PS_GCCC-3′, 5′-GATG_PS_ATCC-3′ and 5′-GGCG_PS_AACGTG-3′ have been deposited with the Protein Data Bank under accession numbers 7CC9, 7CCJ and 7CCD, respectively.

## Supplementary Material

gkaa574_Supplemental_File
