# Supplementary material for: DNA backbone interactions impact the sequence specificity of DNA sulfur-binding domains: revelations from structural analyses
Source: Nucleic Acids Res. 2020 Jul 4;48(15):8755–66. doi: 10.1093/nar/gkaa574 (PMC7470945; doi:10.1093/nar/gkaa574)
Supplement: gkaa574_Supplemental_File [file gkaa574_supplemental_file.pdf]

SUPPLEMENTARY DATA

DNA backbone interactions impact the sequence specificity of DNA sulfur-binding domains: revelations from structural analyses

Table S1. Primers used for construction of protein expression vectors

Table S2. Data collection and refinement statistics for SBD_{Spr} complexes

Table S3. K_D values of SBD_{Spr}-mutant proteins for $G_{PS}GCC$

Table S4. Binging free energy (Units in kcal/mol) between PT-DNA and SBD_{Sco}

Figure S1. Determination of the binding affinity of SBD_{Spr} for R_P and S_P stereoisomers of $G_{PS}GCC$, $G_{PS}ATC$, $G_{PS}AAC$, $G_{PS}TTC$, and $C_{PS}CA$ by fluorescence polarization assay.

Figure S2. Superimposition of SBD_{Spr} with PT-DNA in different core sequences.

Figure S3. Structure of the SBD_{Spr} complexed with PT-DNA.

Figure S4. Determination of the binding affinity of SBD_{Spr}-mutant proteins for $G_{PS}GCC$ through EMSA and fluorescence polarization assay.

Figure S5. Superimposition of the SBD_{Spr}-DNA complex (in cyan and orange) and SBD_{Sco}-DNA complex (in grey and yellow).

Figure S6. Superimposition of the hydrophobic cavity in SBD_{Spr} and SBD_{Sco}.

Figure S7. The ligand-receptor interaction of methyl groups in the T6=A6' and A6=T6' base pairs in $G_{PS}ATC$ and $G_{PS}AAC$.

Figure S8. Determination of the binding affinity of SBD_{Sco} mutants.

Figure S9. Noncovalent interaction (NCI) analysis of the interface domain in SBD_{Sco}-G_{PS}GCC, SBD_{Sco}-G_{PS}ATC and SBD_{Sco}-G_{PS}AAC.

Figure S10. Structural comparison of representative structures of SBD_{Sco}-E156R/D157R and wild type extracted from clustering analysis.

Figure S11. Ability of the SBD_{Sco}-E156R/D157R mutant to bind R_P and S_P stereoisomers of PT-DNA.

Figure S12. Multiple sequence alignment of SBD homologs.

Table S1. Primers and oligonucleotides used in this study

PRIMERS	SEQUENCE
SBDspr-F	GGACCATATGCCGCTCACCGACACAGACCGGT
SBDspr-F	GGAATTCTTAGCCTTCGTCGTACAGGCCGG
SBDspr-Y31A-F	CGCGCCTC GCC CAGCCGATCACTCTGCT
SBDspr-Y31A-R	ATCGGCTGGGCGAGGCGCGGTCCGTTCG
SBDspr-Q32A-F	GCCTCTAT GCG CCGATCACTCTGCTGTG
SBDspr-Q32A-R	GTGATCGGCGCATAGAGGCGCGGTCCGT
SBDspr-Y78A-F	GCCCGGAC GCC CCCGTCCTCGCGCTCCA
SBDspr-Y78A-R	AGGACGGGGGCGTCCGGGCGGGGGCGCT
SBDspr-P79A-F	CGGACTAC GCC GTCCTCGCGCTCCACCG
SBDspr-P79A-R	GCGAGGACGGCGTAGTCCGGGCGGGGGC
SBDspr-A82G-F	CCGTCCTC GGG CTCCACCGCGCGGGGTT
SBDspr-A82G-R	CGGTGGAGCCCGAGGACGGGGTAGTCCG
SBDspr-H102A-F	CCACCGCG GCC GGCGACTCGGCGCTCAG
SBDspr-H102A-R	GAGTCGCCGGCCGCGGTGGGCACCTCGC
SBDspr-G103A-F	CCGCGCAC GCC GACTCGGCGCTCAGGAA
SBDspr-G103A-R	GCCGAGTCGGCGTGCGCGGTGGGCACCT
SBDspr-D104A-F	CGCACGGC GCC TCGGCGCTCAGGAACTG
SBDspr-D104A-R	AGCGCCGAGGCGCCGTGCGCGGTGGGCA
SBDspr-R73A-F	GGGGGGAG GCC CCCCGCCCGGACTACCC
SBDspr-R73A-R	GGGCGGGGGGCCTCCCCCCGGGCCCCGT
SBDspr-R85A-F	CGCTCCAC GCC GCGGGGTTGTGGACGCT
SBDspr-R85A-R	AACCCCGCGGCGTGGAGCGCGAGGACGG
SBDsco-F	GGAC CATATG ATCAGGGAGGCCCCCAAGACCT
SBDsco-R	GGAATTCTTAGTGGTGGTGGTGGTGGTGCGGCCGCAGAGCA TCCGGCCA
SBDsco-E156R-F	GCCAGGTT CGA GACGGTGTGGATGGGGT
SBDsco-E156R-R	ACACCGTCTCGAACCTGGCCGTACTTCT
SBDsco-E156K-F	GCCAGGTT AAG GACGGTGTGGATGGGGT
SBDsco-E156K-R	ACACCGTC CTT AACCTGGCCGTACTTCT
SBDsco-E156L-F	GCCAGGTT TTA GACGGTGTGGATGGGGT
SBDsco-E156L-R	ACACCGTC TAA AACCTGGCCGTACTTCT
SBDsco-E156Q-F	GCCAGGTT CAA GACGGTGTGGATGGGGT
SBDsco-E156Q-R	ACACCGTC TTG AACCTGGCCGTACTTCT
SBDsco-E156D-F	GCCAGGTT GAT GACGGTGTGGATGGGGT
SBDsco-E156D-R	ACACCGTC ATC AACCTGGCCGTACTTCT
SBDsco-D157R-F	CAGGTTGAAAGGGGTGTGGATGGGGTGCG
SBDsco-D157R-R	ATCCACACCCCTTTCAACCTGGCCGTACT
SBDsco-D160R-F	GTTGAAGACGGTGTGAGAGGGGTGCGCTACCCATTCTGGGC
SBDsco-D160R-R	TGGGTAGCGCACCCCTCTCACACCGTCTTCAACCTGGCCGT
SBDsco-E156R/D157R-F	CAGGTTCGAAGGGGTGTGGATGGGGTGCG
SBDsco-E156R/D157R-R	ATCCACACCCCTTCGAACCTGGCCGTACT

**OLIGONUCLEOTIDES**	**SEQUENCE**
PT-GGCC-8	5′-GGCG$_{PS}$GCCC-3′
	3′-CCGCCGGG-5′
PT-GGCC-10	5′-CCCG$_{PS}$GCCGCC-3′
	3′-GGGCCGGCGG-5′
GGCC-10	5′-CCCGGCCGCC-3′
	3′-GGGCCGGCGG-5′
PT-GATC-8	5′-GATG$_{PS}$ATCC-3′
	3′-CTACTAGG-5′
PT-GATC-10	5′-GATG$_{PS}$ATCCTA-3′
	3′-CTACTAGGAT-5′
GATC-10	5′-GATGATCCTA-3′
	3′-CTACTAGGAT-5′
PT-GAAC-10	5′-GGCG$_{PS}$AACGTG-3′
	3′-CCGCTTGCAC-5′
GAAC-10	5′-GGCGAACGTG-3′
	3′-CCGCTTGCAC-5′
PT-GTTC-10	5′-GGCGAACGTG-3′
	3′-CCGCTT$_{PS}$GCAC-5′
PT-CCA-10	5′-GAACC$_{PS}$CAAGC-3′
	3′-CTTGGGTTCG-5′
CCA-10	5′-GAACCCAAGC-3′
	3′-CTTGGGTTCG-5′

Table S2. Data collection and refinement statistics for SBD_{Spr} complexes

	**SBD_{Spr}-G_{PS}GCC complex**	**SBD_{Spr}-G_{PS}ATC complex**	**SBD_{Spr}-G_{PS}AAC complex**
**Data collection**			
Beamline	BL19U1	BL19U1	BL18U1
Wavelength (Å)	0.97925	0.97925	0.97776
Resolution range*	48.13 - 2.063 (2.137 - 2.063)	31.12 - 3.3 (3.417 - 3.3)	28.63 - 2.42 (2.506 - 2.42)
Space group	$P6_122$	$C222_1$	$P1$
Unit cell parameters			
a, b, c (Å)	154.5, 154.5, 156.8	97.7, 105.3, 116.4	46.94, 48.94, 56.19
α, β, γ (°)	90, 90, 120	90, 90, 90	107.04, 109.79, 97.26
Unique reflections*	68202 (6703)	9259 (922)	13319 (1393)
Completeness (%)*	100(100)	100(100)	80 (85)
$I/\sigma I$*	32.95 (1.53)	6.5 (1.5)	80.29 (1.47)
Redundancy*	38.3(29.8)	13.1 (13.9)	3.5 (3.5)
R-merge (%)*	14.2 (247.7)	45.0 (155.1)	7.4 (52.7)
**Refinement**			
Resolution range	48.13 - 2.063	31.12 - 3.3	28.63 - 2.42
Average B-factor	36.12	62.13	47.80
R_{work}/R_{free} factors (%)	20.98/23.04	24.47/29.54	19.99/24.77
RMSD bond lengths (Å)	0.005	0.006	0.007
RMSD bond angles (º)	0.75	0.75	0.97
Ramachandran plot (favoured, allowed, outliers, %)	98, 1.9, 0	93, 6.3, 0.32	98, 1.8, 0.61
PDB code	7CC9	7CCJ	7CCD

*Statistics for the highest-resolution shell are shown in parentheses.

R-merge = $\sum|I - \langle I \rangle|/\sum I$, where I is the observed intensity and $\langle I \rangle$ is the averaged intensity from multiple observations.

$\langle I/\sigma I \rangle$ = averaged ratio of the intensity (I) to the error of the intensity (σI).

Rwork = $\sum|F_{obs} - F_{cal}|/\sum|F_{obs}|$, where F_{obs} and F_{cal} are the observed and calculated structure

factors, respectively.

R_{free} was calculated using a randomly chosen subset (5%) of the reflections not used in refinement.

RMSD, root-mean-square deviation from ideal geometry. Data for the highest resolution shell are shown in parentheses.

Abbreviations: PDB, Protein Data Bank; RMSD, root mean square deviation.

Table S3. K_D values of SBD_{Spr}-mutant proteins for $G_{PS}GCC$

SBD_{Spr} mutant	K_D (nM)
(wild type)	5.6±0.9
Y31A	>3156
Q32A	279±49
Y78A	>4475
P79A	67±8
H102A	>1857
G103A	>9000
D104A	144±12
R73A	>1252
R85A	31±2

Table S4. Binging free energy (Units in kcal/mol) between PT-DNA and SBD_{Sco}

System	ΔE_{vdW}	ΔE_{ele}	ΔG_{GB}	ΔG_{SA}	ΔG_{bind}	$\Delta\Delta G_{bind}$
SBD_{Sco}	-98.6±7.4	-1260.9±79.8	1288.4±76.6	-13.3±0.8	-84.4±4.4	0
SBD_{Sco}-E156R/D157R	-76.2±7.5	-1207.7±79.8	1212.5±76.6	-11.1±0.8	-83.5±4.8	0.9±0.4

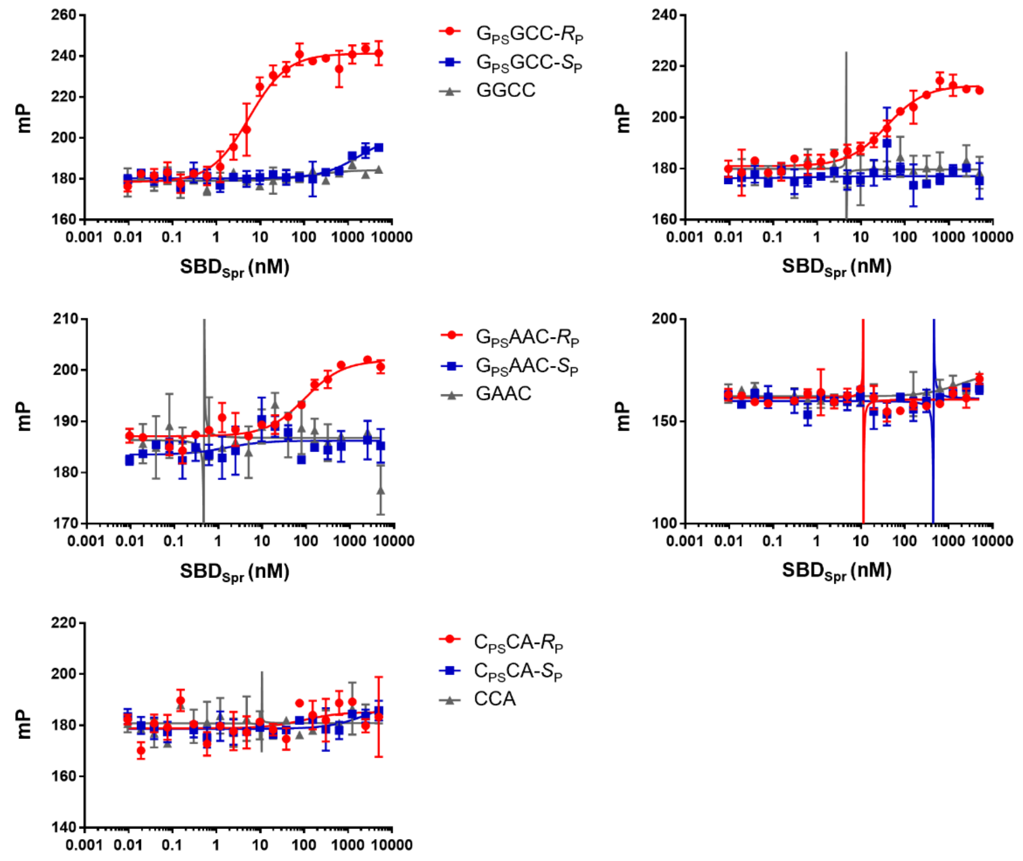

Figure S1. Determination of the binding affinity of SBD_{Spr} for R_P and S_P stereoisomers of $G_{PS}GCC$, $G_{PS}ATC$, $G_{PS}AAC$, $G_{PS}TTC$, and $C_{PS}CA$ by fluorescence polarization assay.

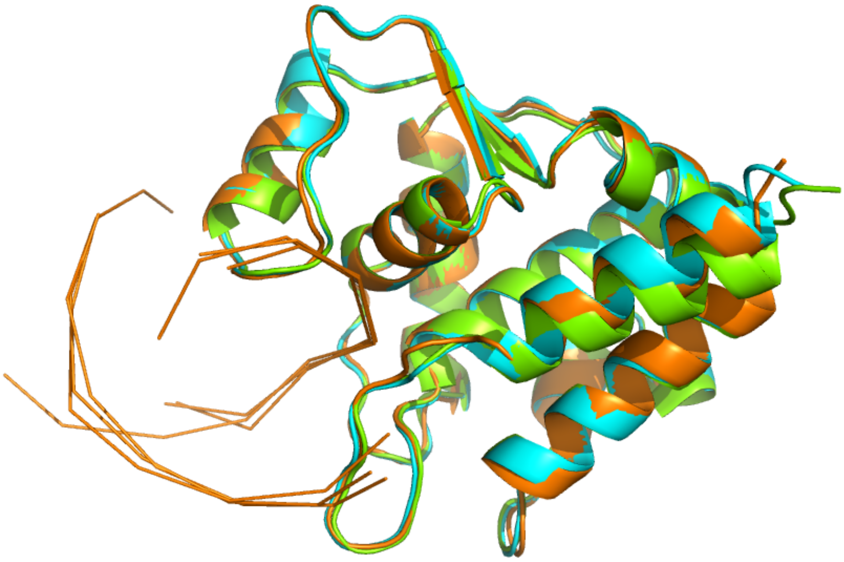

Figure S2. Superimposition of SBD_{Spr} with PT-DNA of different core sequences. SBD_{Spr}-G_{PS}GCC, SBD_{Spr}-G_{PS}ATC, and SBD_{Spr}-G_{PS}AAC are colored in cyan, orange, and green, respectively.

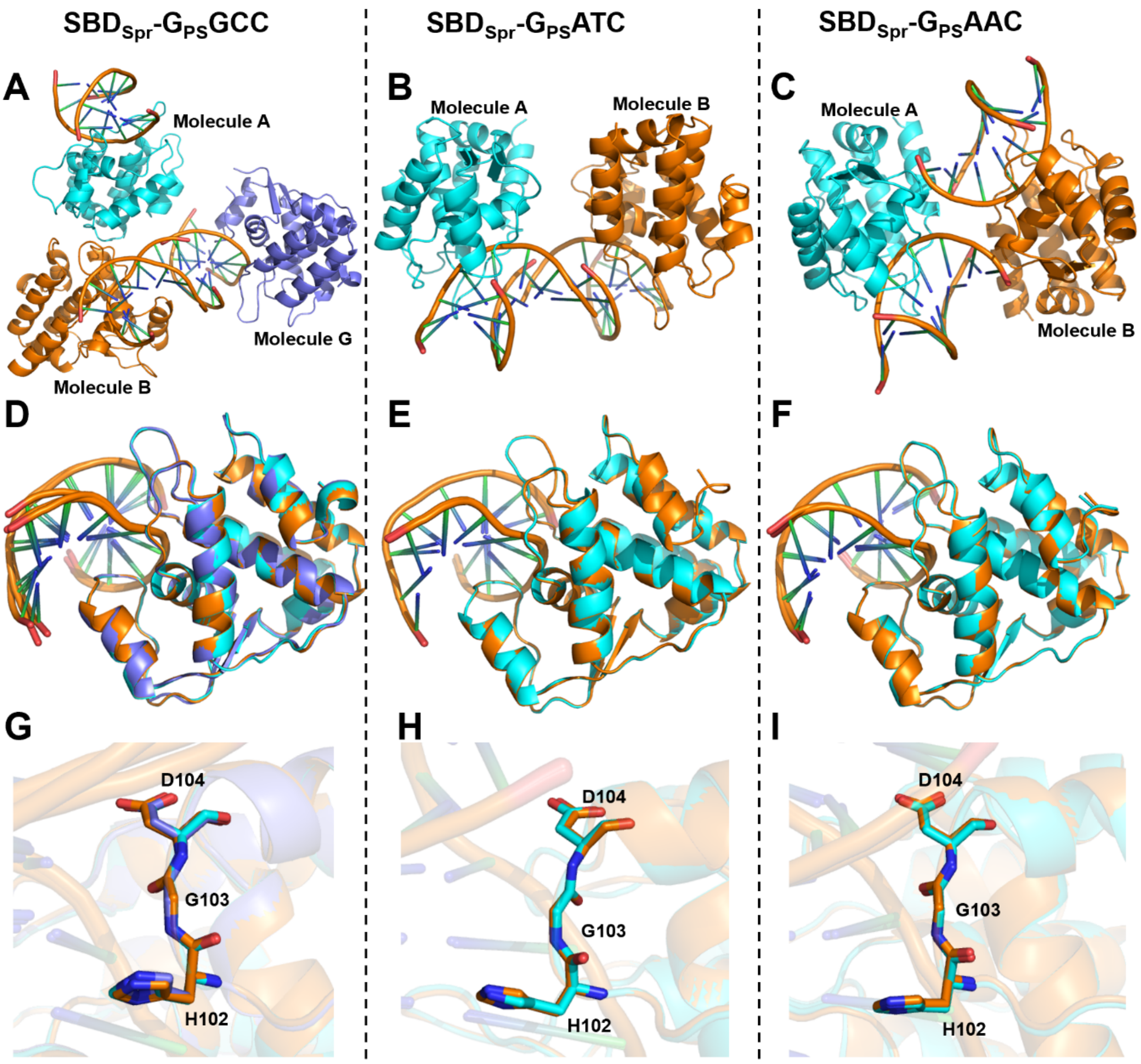

Figure S3. Structures of the SBD_{Spr} complexed with PT-DNAs. **(A)** Trimer form of the SBD_{Spr}-$G_{PS}GCC$ complexes. **(B)** Dimer form of the SBD_{Spr}-$G_{PS}ATC$ complexes. **(C)** Dimer form of the SBD_{Spr}-$G_{PS}ACC$ complexes. **(D)** Superimposition of molecule A, B, and G of SBD_{Spr}-$G_{PS}GCC$ complexes, yielding an RMSD of 0.140 Å. **(E)** Superimposition of molecule A and B of SBD_{Spr}-$G_{PS}ATC$ complexes, yielding an RMSD of 0.167 Å. **(F)** Superimposition of molecule A and B of SBD_{Spr}-$G_{PS}AAC$ complexes, yielding an RMSD of 0.250 Å. **(G, H, I)** Superimposition of HGD loop in different molecule of SBD_{Spr}-$G_{PS}GCC$ complexes, SBD_{Spr}-$G_{PS}ATC$ complexes and SBD_{Spr}-$G_{PS}AAC$ complexes, respectively.

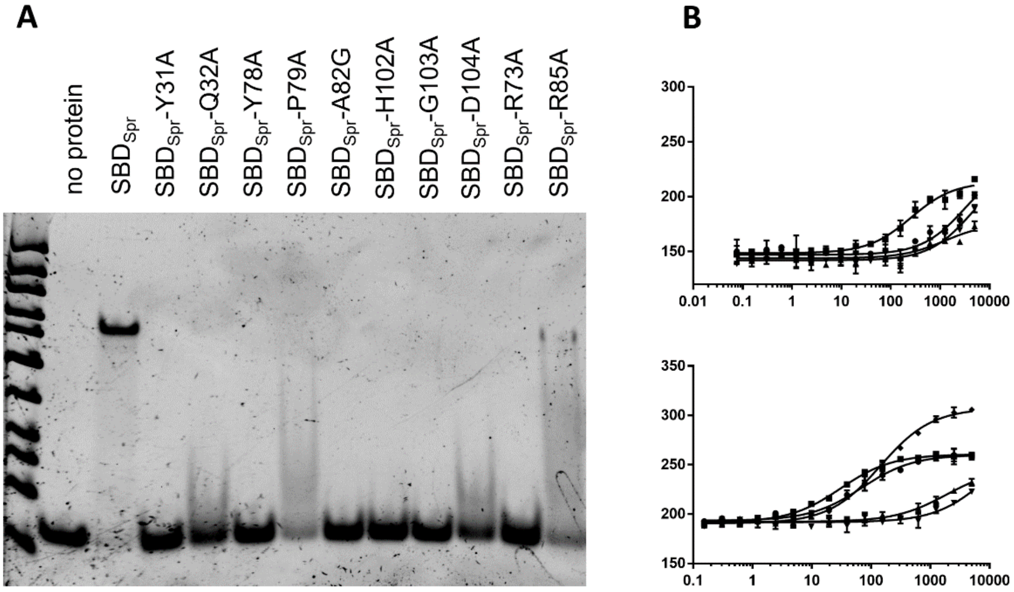

Figure S4. Determination of the binding affinity of SBD_{Spr} mutant proteins for $G_{PS}GCC$ through **(A)** EMSA and **(B)** fluorescence polarization assay.

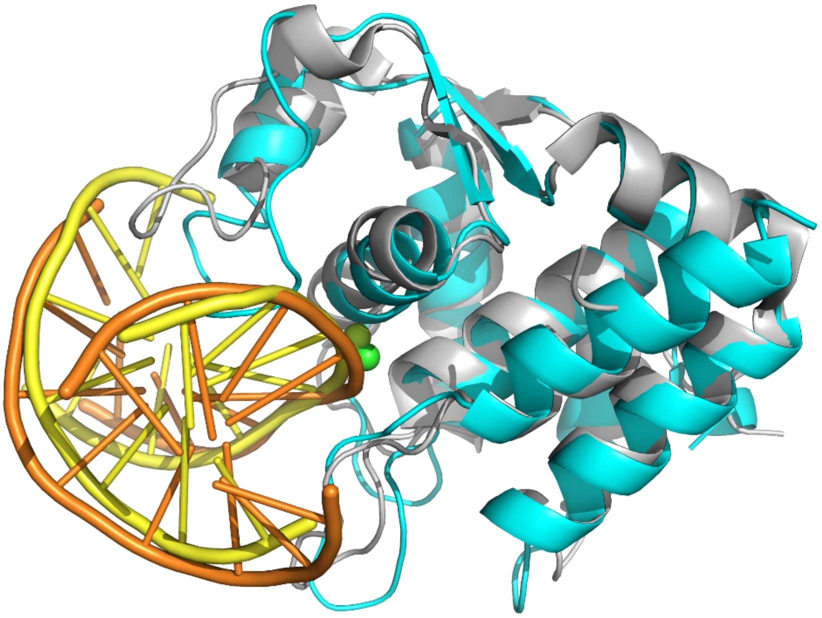

Figure S5. Superimposition of the SBD_{Spr}-DNA complex (in cyan and orange) and SBD_{Sco}-DNA complex (in grey and yellow).

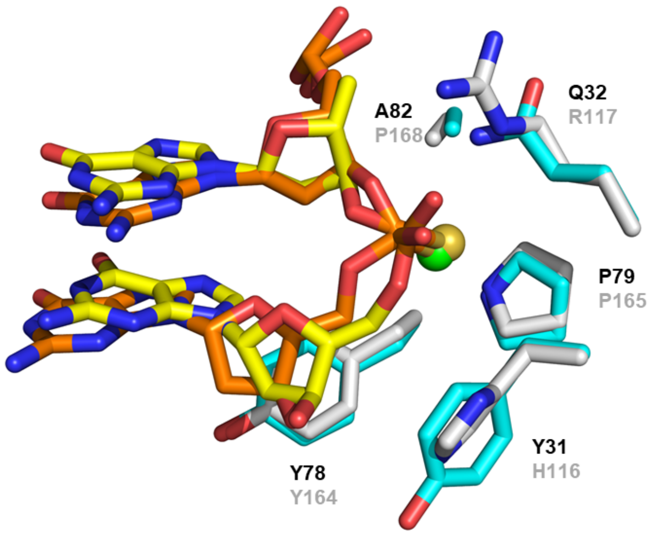

Figure S6. Superimposition of the hydrophobic cavity in SBD_{Spr} (cyan) and SBD_{Sco} (grey).

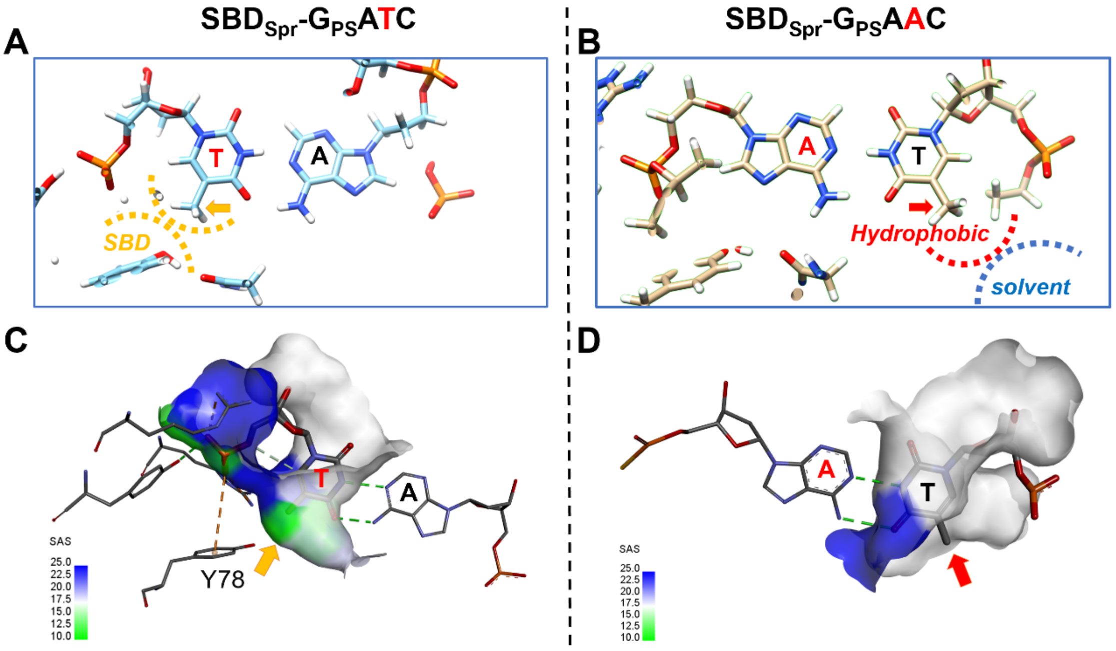

Figure S7. The ligand-receptor interaction of methyl groups in the T6=A6' and A6=T6' base pairs in G_{PS}ATC and G_{PS}AAC. (A, B) Schematic diagram of the hydrophobic interaction in SBD_{Spr}-G_{PS}ATC and SBD_{Spr}-G_{PS}AAC complex. (C, D) The solvent accessibility surface (SAS) of T^6 in SBD_{Spr}-G_{PS}ATC and $T^{6'}$ SBD_{Spr}-G_{PS}AAC complex, using the percent solvent accessibility in a scale of 10-25 for a better color contrast. Surface was colored by the solvent accessibility of the receptor residues from blue for exposed to green for buried. The arrows pointed to the methyl group of thymine.

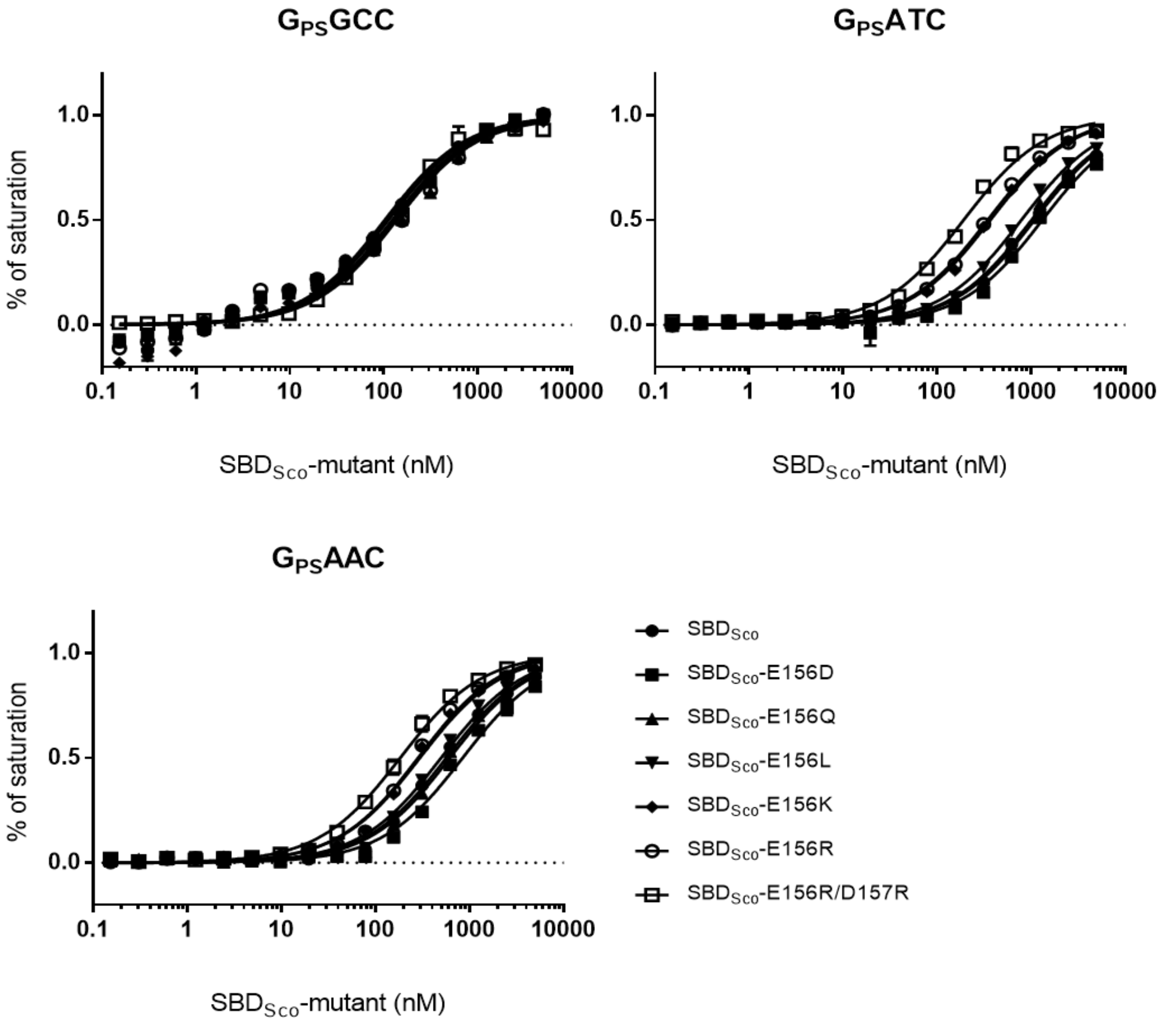

Figure S8. Determination of the binding affinity of SBD_{Sco} mutants by fluorescence polarization assay. Mutations were generated at sites E156 and D157 in SBD_{Sco}, and the binding affinities of the resulting mutants for G_{PS}GCC, G_{PS}ATC, and G_{PS}AAC were calculated and are listed in Table 2.

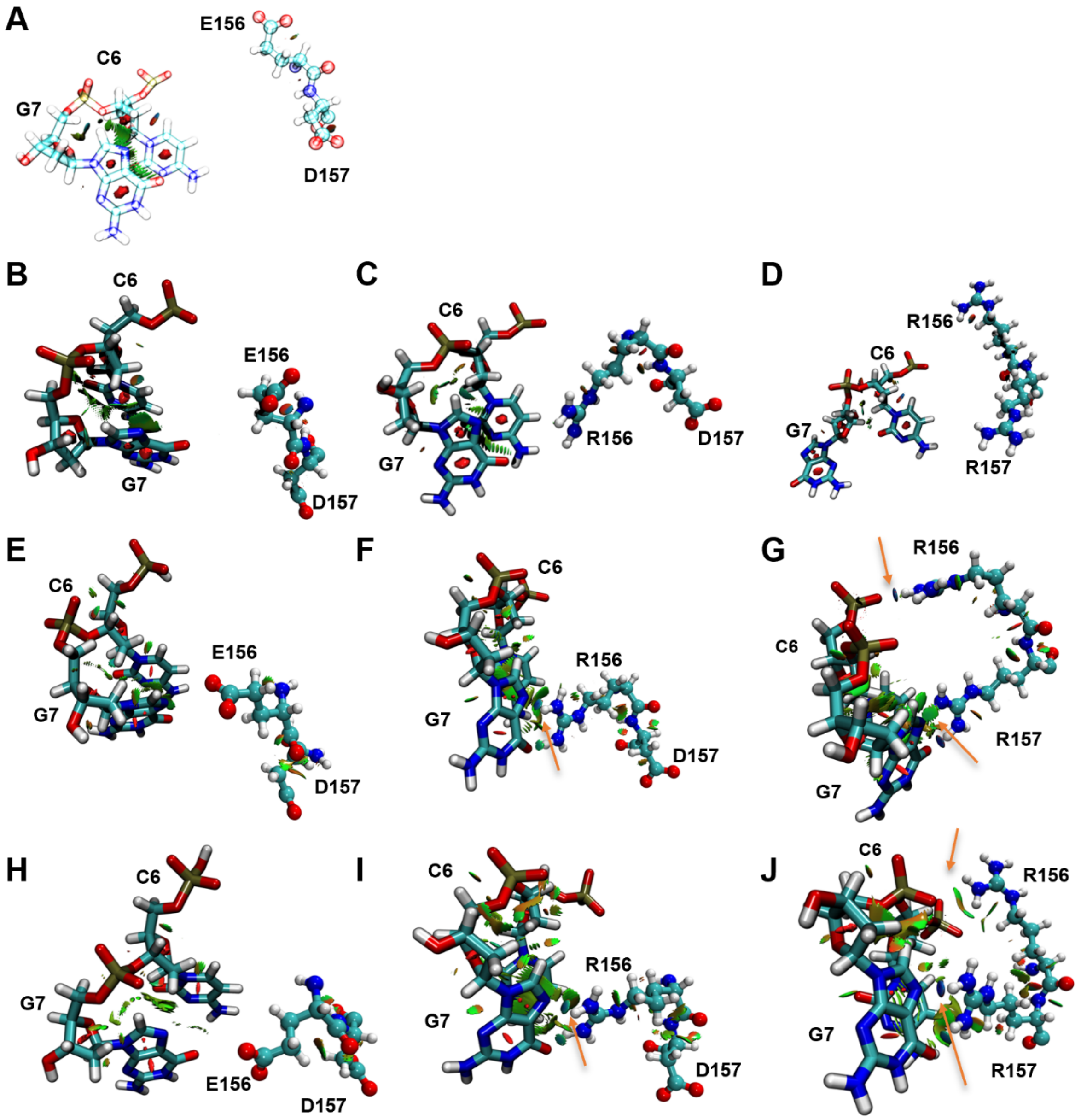

Figure S9. Noncovalent interaction (NCI) analysis of the interface domain in SBD_{Sco}-$G_{PS}GCC$, SBD_{Sco}-$G_{PS}ATC$ and SBD_{Sco}-$G_{PS}AAC$ using reduced density gradient (RDG) method. (A) SBD_{Sco}-$G_{PS}GCC$ crystal structure, showed as transparent. Panel (B) wild-type SBD_{Sco}; (C) SBD_{Sco}-E156R mutant; (D) SBD_{Sco}-E156R/D157R mutant in complex with $G_{PS}GCC$ after MD simulations. Panel (E) wild-type SBD_{Sco}; (F) SBD_{Sco}-E156R mutant; (G) SBD_{Sco}-E156R/D157R mutant in complex with $G_{PS}ATC$ after MD simulations. Panel (H) wild-type SBD_{Sco}; (I) SBD_{Sco}-E156R mutant; (J) SBD_{Sco}-E156R/D157R mutant in complex with

$G_{PS}AAC$ after MD simulations. Plots are given with an isovalue of 0.05 a.u. Arrows indicate the interaction regions between nucleotides and amino acid residues. RDG isosurfaces were colored according to the value of sign(λ_2) ρ, where a BGR (blue-green-red) color scale was adopted. Blue color represents attractive or bonding interaction, green weak van der Waals interaction, and red repulsive interaction. All isosurfaces are colored according to a BGR scheme over the electron density range -0.05 a.u. < sign(λ_2) ρ < 0.05 a.u..

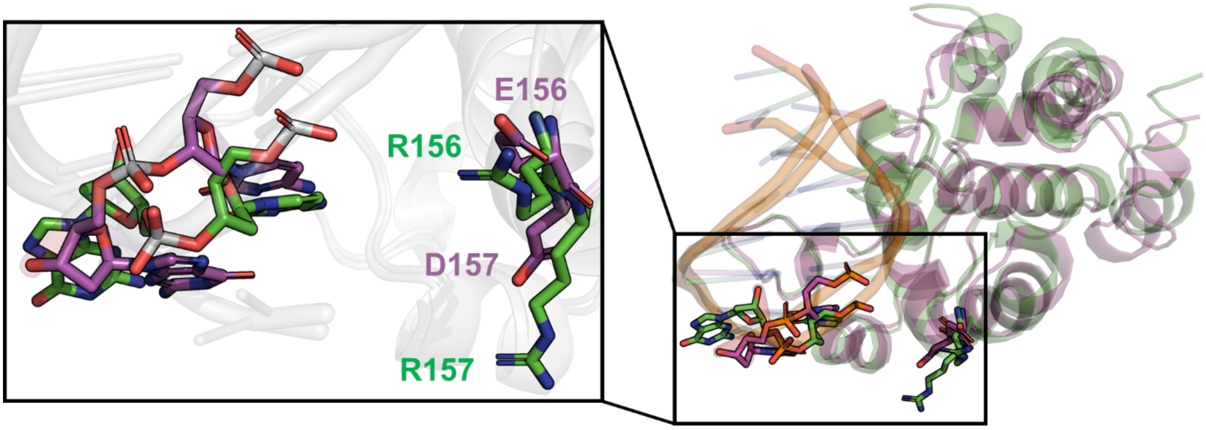

Figure S10. Structural comparison of representative structures of SBD_{Sco}-E156R/D157R and wild type extracted from clustering analysis. Superposition of SBD_{Sco}-E156R/D157R structure (green) with wild type (purple).

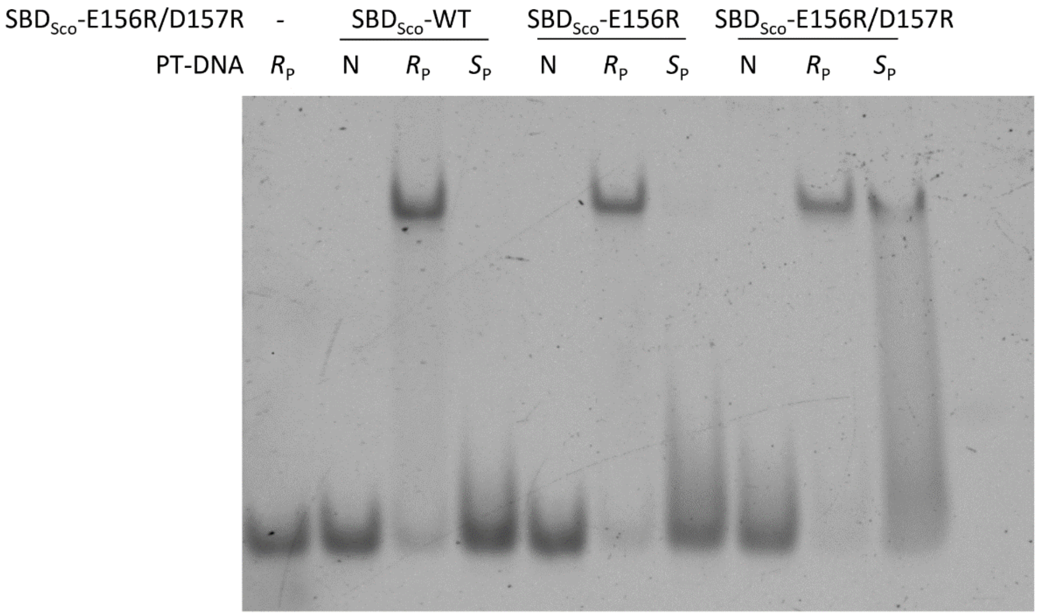

Figure S11. Ability of the SBD_{Sco}-E156R/D157R mutant to bind R_P and S_P stereoisomers of hemi-PT-DNA. A 10 bp PT-DNA oligonucleotide (5′-CCCG$_{PS}$GCCGCC-3′) was used as the DNA substrate in the EMSA. N, DNA oligonucleotide with no modification; R_P, R_P stereoisomers of hemi-PT-DNA; S_P, S_P stereoisomers of hemi-PT-DNA.

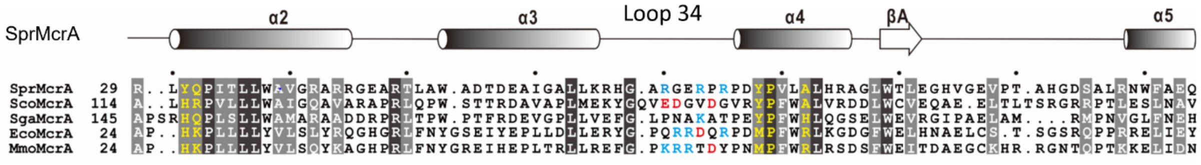

Figure S12. Multiple sequence alignment of SBD homologs. SprMcrA from *Streptomyces pristinaespiralis* was aligned with homologs from *Streptomyces coelicolor* (ScoMcrA), *Streptomyces gancidicus* (SgaMcrA), *Escherichia coli* (EcoMcrA), and *Morganella morganii* (MmoMcrA). The basic amino acid residues of loop 34 are highlighted in blue, and acidic amino acid residues are highlighted in red. The sulfur-recognizing residues are colored in yellow. Shading indicates conserved residues. The secondary structural features of SprMcrA are shown above the alignment.
